# Global Groundwater Solute Composition and Concentrations

**DOI:** 10.1111/gwat.13205

**Published:** 2022-05-26

**Authors:** Warren W. Wood, Pauline L. Smedley, Bruce D. Lindsey, Warren T. Wood, Roberto E. Kirchheim, John A. Cherry

**Affiliations:** ^1^ Department of Earth and Environmental Sciences Michigan State University 206 Natural Sciences Building, 288 Farm Lane, East Lansing MI 48824 USA; ^2^ British Geological Survey Nicker Hill, Keyworth, Nottingham NG12 5GG UK; ^3^ U.S. Geological Survey 215 Limekiln Road, New Cumberland PA 17070 USA; ^4^ Geology and Geophysics Section Naval Research Laboratory NRL Code 7432, John C. Stennis Space Center MS 39529 USA; ^5^ Program for Isotope and Hydrochemistry Geological Survey of Brazil (CPRM‐SGB) Rua Costa, 55—Cerqueira César, São Paulo CEP 01304‐010 Brazil; ^6^ Morwick G360 Institute for Groundwater Research University of Guelph 50 Stone Road East, Thornbrough Building, Guelph Ontario N1G 2W1 Canada

## Abstract

Informed analysis of policies related to food security, global climate change, wetland ecology, environmental nutrient flux, element cycling, groundwater weathering, continental denudation, human health, and others depends to a large extent on quantitative estimates of solute mass fluxes into and out of all global element pools including the enigmatic global aquifer systems. Herein for the first time, we proffer the mean global solute concentration of all major and selected minor and trace solutes in the active groundwater that represents 99% of liquid fresh water on Earth. Concentrations in this significant element pool have yielded to a geospatial machine learning kNN‐nearest neighbors' algorithm with numerous geospatial predictors utilizing a large new lithology/climate/aquifer age/elevation based solute database. The predicted concentrations are consistent with traditional solute ratios, concentrations, and thermodynamic saturation indices.

## Introduction

Enigmatic fresh groundwater systems represent 99% of liquid fresh water on Earth (Shiklomanov 1993) and supplies approximately 70% of all human use (Wood and Cherry 2021) thus, it is of crucial importance. Policies related to global climate change, food security, environmental nutrient flux, global element cycling, wetland ecology, and human health depend to a large extent on quantitative estimates of mass fluxes into and out of all global element pools. Estimates of mass flux into and out of the terrestrial element pools require knowledge of both water fluxes and solute concentrations. We have well‐established global values of freshwater fluxes including global precipitation (Huffman et al. 2001, 2009; Adler et al. 2003); global evapotranspiration (Wang and Dickinson 2012); global river flow (Dai and Trenberth 2002); and global active aquifer volume (Gleeson et al. 2016). Previous estimates of the global solute concentration of freshwater have been limited to rivers (Clark 1924; Livingston 1963 and Meybeck 2003). Herein we present mean and median concentrations of all major and selected minor and trace solutes from the global active aquifer systems. We anticipate that these data will be utilized in much the same as the global riverine values are used (Klee and Graedel 2004; Sen and Peucker‐Ehrenbrink 2012).

Groundwater solute chemistry is naturally highly variable on a local, regional, and global scale. Solute concentrations vary over one to two orders of magnitude and there are several different solute types (sodium chloride, sodium bicarbonate, calcium bicarbonate, etc.) depending on the lithology of surrounding rocks, atmospheric input, discharge of underlying brines, and legacy solutes. Thus, to obtain unbiased global mean and median values we first created a globally representative solute database that includes an extremely wide range of aquifer lithology, age, topography, climatic, and hydrologic conditions that were used in a geospatial machine learning (GML) algorithm with geospatial predictors to calculate a mean and median global value for each solute. Finally, we evaluated the predicted values by assessing their geochemical ratios thermodynamic saturation, and other hydrogeochemical indicators.

## Active Aquifer Solute Database

Active freshwater aquifers systems are defined herein as having received recharge within approximately the last 100 years that typically contains dissolved solids less than 500 mg/L, solute ratios typical of human consumption, and in general have a well depth of less than 500 m. Based on the typical length of flow lines, hydraulic conductivity, and gradient, active freshwater aquifers systems typically exhibit a mix of low flux old (thousands of years); intermediate flux of medium age (hundreds of years); and a large flux of young water (<100 years). Active groundwater typically exhibits adjusted carbon‐14 ages of hundreds to thousands of years and yet contain recent chlorofluorocarbons, tritium, or other solutes consistent with recent recharge, thus, a mix of water of different age consistent with the well‐known exponential age distribution (Starn et al. 2021). The assertion that the samples represent active aquifer systems is supported by the observation that of the 3493 samples analyzed for tritium in the U.S. Geological Survey's NAWQA database, 2702 were positive (Lindsey et al. 2019). As most tritium is generated by cosmic ray‐induced spallation of nuclides; residual activities from atmospheric nuclear weapons testing; and ongoing nuclear fuel‐cycle operations thus, the presence of tritium in groundwater is consistent with recent atmospheric contact. It is assumed that this NAWQA database is representative of our other national databases.

To represent the elemental pool of active aquifer systems we assembled a large, globally representative solute database from approximately 24,000 wells with nearly 267,000 individual analytical values from the Australian Global Explorer (*n* = 15,016); the Geological Survey of Brazil (*n* = 3255; de Informações de Águas Subterrâneas); the Geological Survey of Chile (*n* = 74; Banco Nacional de Aguas); and the U.S. Geological Survey (*n* = 5471; DeSimone et al. 2014; Arnold et al. 2016, 2017, 2018, 2020). Samples in this database represent an extremely wide range of aquifer ages from Precambrian to Holocene, all globally significant lithologic/depositional environments, most redox and pH conditions, and a full range of climates from hyper‐arid to tropical. The locations exhibited an extensive range of land‐use practices presumed by the owners and operators to be generally free of known point sources of anthropogenic contaminants, Analyses with cation/anion imbalance greater than 5% (Hem 1985) were rejected as were samples with chloride values >19,000 mg/L and aquifers with temperature >40 °C. We also exclude known brines and geothermal sources from our discussion as in general they have solute concentrations greater than most human needs and are not significantly involved with surface or near‐surface environmental processes (Appendix S3). Note that it was beyond the scope of this study to distinguish between solutes in surficial, unconfined, or confined active aquifer systems.

Owing to the wide range of environments and lithologic types we believe our current database provides an adequate input to the geospatial machine language algorithm; we did, however, attempt to expand our data search to other continents. The search for additional national solute databases resulted in many that were not publicly available; some required purchase; some were poorly curated with many improbable values; some nations ignored our requests; some comingled a mix of private confidential and government data and could not be released; some were collected largely for contamination studies; some did not distinguish between uses and comingled groundwater analyses including oil filed brines and waste injection and fracking wells, and some covered only a limited range of climate/hydrology.

**Figure 1:**
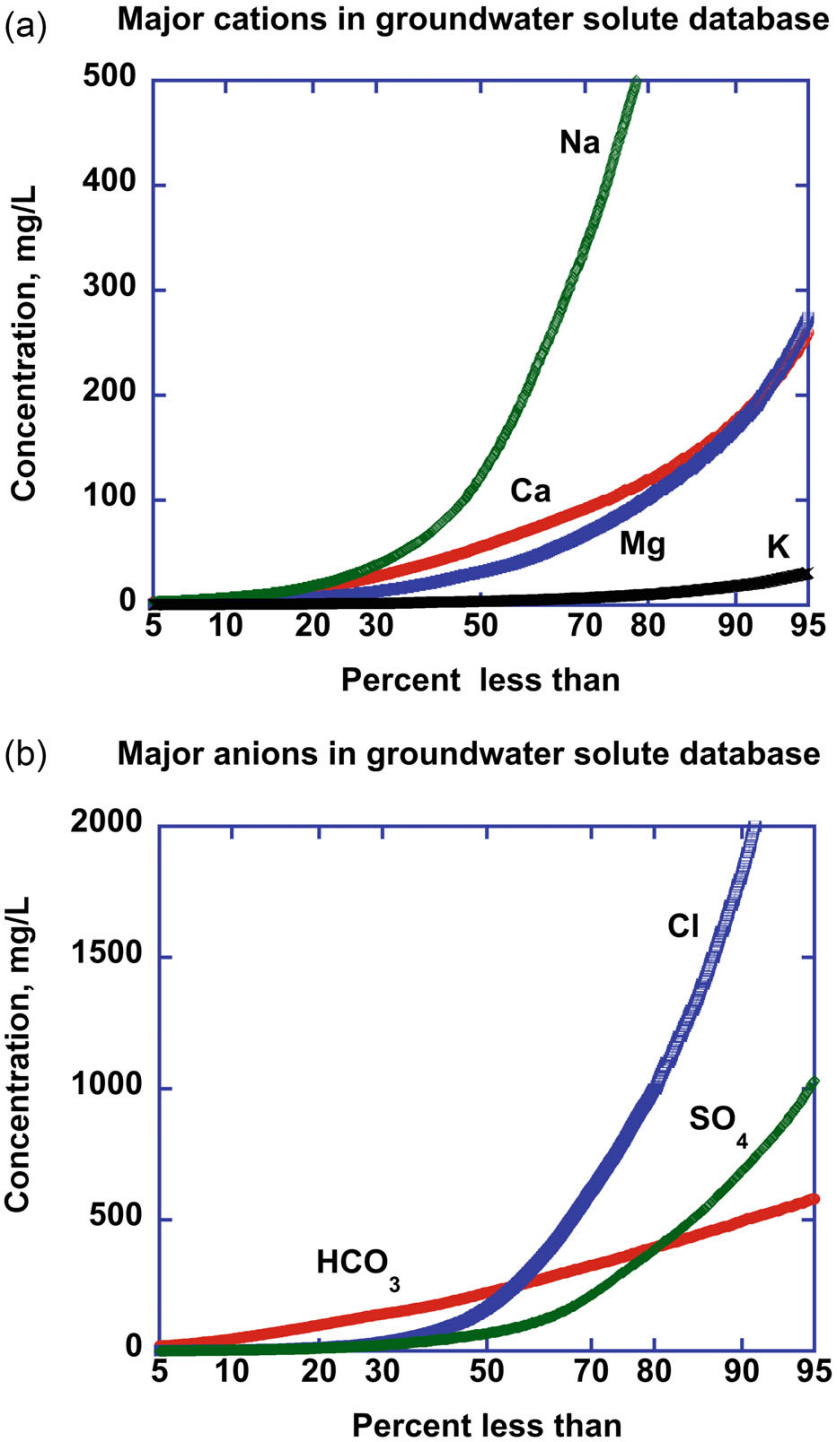
(a) Solute database cumulative frequency within the 5–95 percentile range of major cations, in mg/L. –(b) Cumulative frequency within the 5–95 percentile range of major anions, in mg/L. Note the different vertical concentration scales between the two figures.

Trace elements (less than 0.1% of total dissolved solids) such as As, Cd, Cr, Mo, Pb, Rd, U, and others are extremely important in evaluating impacts on human health; however, their concentrations are dependent on local mineralogy and pH/Eh conditions that are highly variable both within and between aquifers, are highly dependent on analytical methods and the number of analyses available in each major lithology/climate/age environment is small thus, are not included in our global database. The trace elements Fe and Sr were included because both are ubiquitous and there existed a large database that included all major lithology/climate/age/elevation environments. Maps showing the distribution of samples and tables of a statistical summary of the database are provided in Appendix S1. The cumulative frequency distribution of major ions from this solute database illustrates the significantly different concentrations between the individual solutes and the lack of normal distributions, except for HCO_3_ (Figure 1a and 1b).

To assess if our solute database was representative of global elemental pools, we compared the mean bicarbonate concentration in our solute database (257 mg/L ± 5% [244–270 mg/L] (Table 1‐SI; Appendix S1) to the mean of 242 ± 5% (230–254 mg/L) of 7395 independent analyses from areas not in our solute database (Algeria, Canada, China, Costa Rico, Egypt, India, Mexico, South Africa, and the UK) (Table 3‐SI; Appendix S1). Bicarbonate ion was chosen to test the data set as it typically constitutes more than half of the total solute mass in active aquifer systems and is approximately normally distributed (Figure 1b) thus, the mean and median are nearly the same. Owing to the lack of published individual values we could not conduct a formal “Student *t* test”; however, it is clear that the mean values overlap at the ±5% uncertainty level. Thus, we conclude that our solute database is likely representative of the global active aquifer solutes and was suitable for input to the kNN algorithm.

### Geospatial Machine Learning

Our database includes all major environments and lithologic types but is unevenly distributed over the Earth's land area; therefore, the GML kNN (*k* nearest neighbor) technique of Fix and Hodges (1951) was used to predict each global solute concentration. For our single global average concentration of each solute, we integrate over each predicted solute grid weighted by the area of each cell. This weighted integration of the prediction mitigates bias in both the geographic and geologic distribution of the sparse samples. The solute input to an analysis by the GML algorithm utilized the “less than 95%” and greater than 5% range of data of each parameter. That is, we assumed the highest and lowest 5% values of the database were not representative of the active aquifer flow zone.

The kNN method used here is described in Lee et al. (2019) to predict seafloor organic carbon concentration and is modified for the application to aquifer solute concentrations (Appendix S3). A variety of machine learning algorithms are available in our code, which uses the Python scikit‐learn library. Most algorithms typically yield similar results for any given application, but the kNN requires the least amount of interpretive input parameters, making it the most data‐driven technique. Fundamentally, each method searches for, and identifies correlations between features and data, then uses those correlations to make predictions. Because our emphasis in this analysis was to mitigate bias in our data and prevent it from propagating to our global estimate, we chose kNN. Its relatively small number of input variables reduces the possibility of bias from interpretive tuning of the input variable. Based on a comparison of algorithms conducted by Graw et al. (2021) on a global, albeit different dataset, we expect the performance of kNN to be similar to other ML algorithms. kNN finds correlations between sparsely sampled observed values and aspects or features of the environment we know (or can accurately estimate) elsewhere. The features are gridded values of quantities that represent what we know about the environment that are potential explanatory variables—topography, sediment type, rock type, average climate, and so on. kNN then uses these correlations to make predictions of values where no values were directly observed. The overarching assumption is that one or more features of the environment correlate with a given observed global quantity, such as solute concentration.

The kNN process includes the following steps: observation gridding, feature generation, feature selection, validation, and prediction. In our implementation of kNN, the observations and features must both exist and be registered to the same grid. In general, the kNN is agnostic to grid orientation or cell size, but for this analysis, we chose a cell (pixel) centered grid of 5 × 5 arc‐minutes in latitude and longitude (about 10 × 10 km at the equator). After gridding, cells containing three or more observed values are represented by the median of those values, cells containing exactly two observed values are represented by the mean of those two values, and cells with a single observed value are represented by that value. Cells with no observed values are the cells for which predicted values are generated.

**Figure 2:**
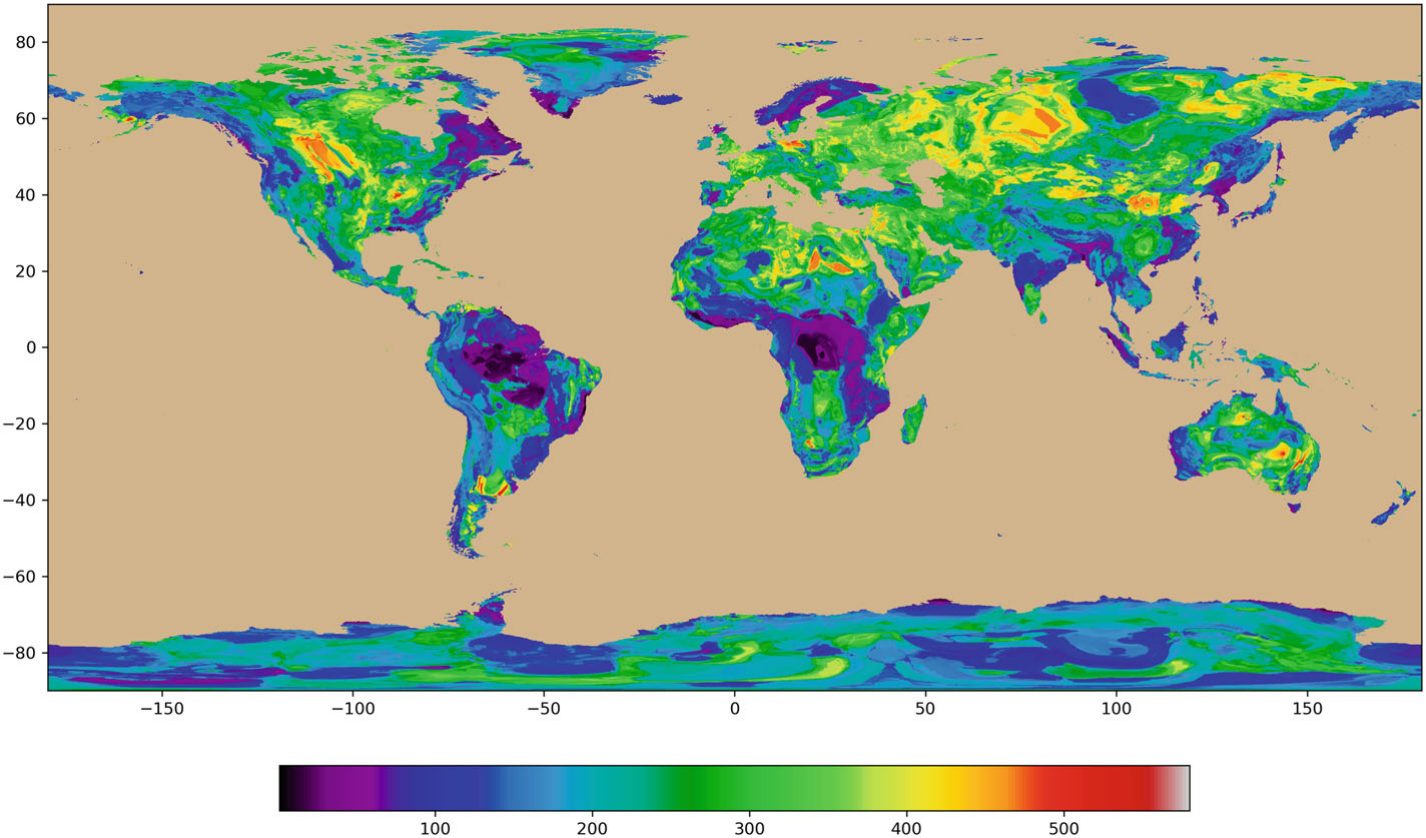
Map of calculated global bicarbonate concentration distribution in active aquifer systems. Vertical scale degrees latitude, horizonal scale degrees longitude, and concentrations in mg/L are given in color. Maps of the other parameters are given in Appendix S2.

**Figure 3:**
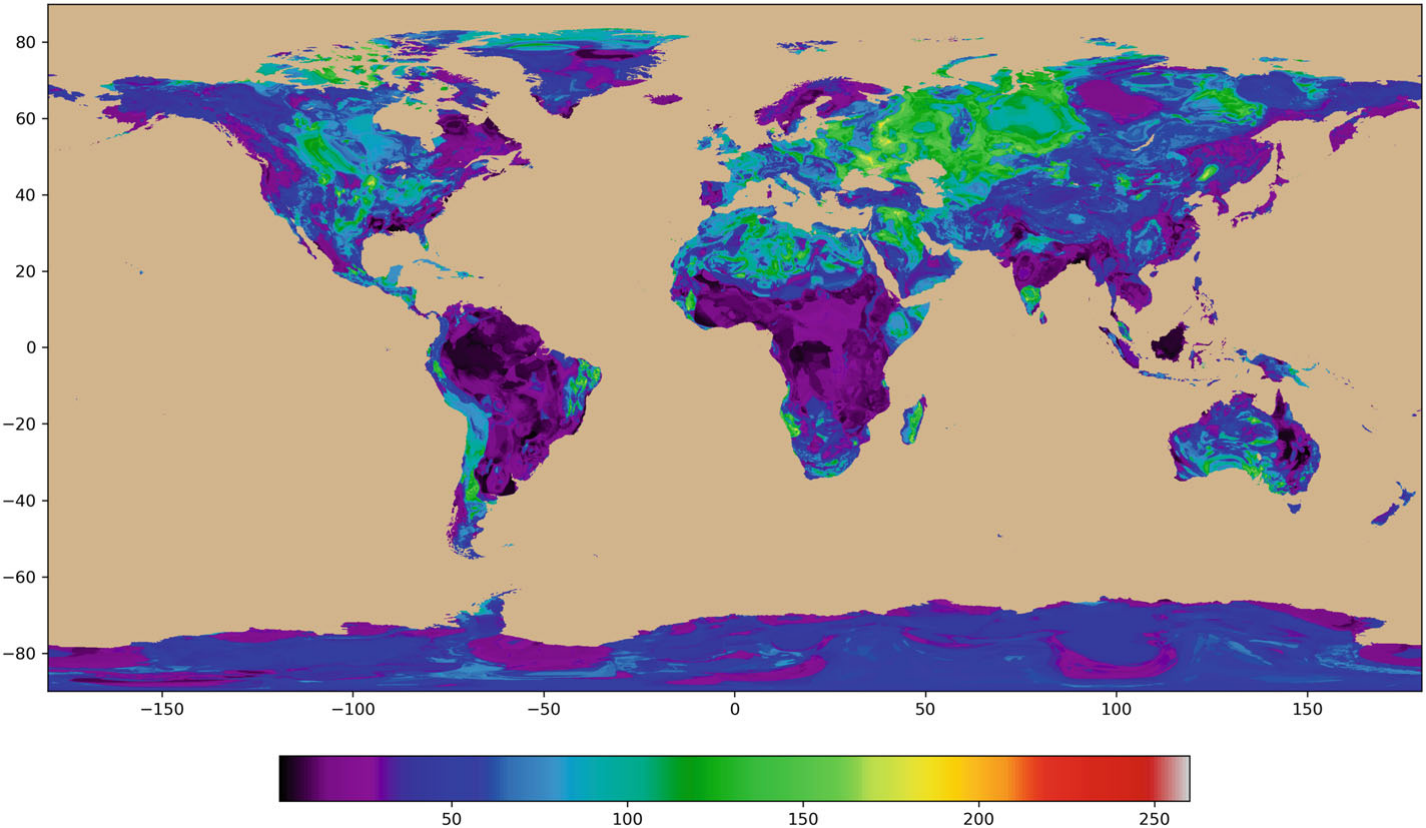
Map of calculated global calcium concentration distribution in active aquifer systems. Vertical scale degrees latitude, horizonal scale degrees longitude, and concentrations in mg/L are given in color. Maps of the other parameters are given in Appendix S2.

Features of the environment must have values at all cells where a prediction is to be made, we analyzed terrestrial not oceanic cells. The features in the analysis come from several publicly available sources. Elevation data were obtained from SRTM15+ (Tozer et al. 2019). The topographic value at each cell was taken to be the median of the 20 × 20, 15‐arc‐second cells in each 5‐arc‐minute cell. Climate data were obtained from the World Climate Database (2021) and included annual averages, minima, maxima, and standard deviations of temperature, precipitation, solar radiation, wind speed, and water vapor pressure, all at 5 × 5 arc‐minutes. Another set of features was generated from a digital geological map produced by Hartmann and Moosdorf (2012). The provinces in this map were descriptive (e.g., metamorphic). To be useful for our analysis, each of the thirteen rock types was converted to a fraction of dominant element types (silica, calcium, iron, and potassium) following Turekian and Wedepohl (1961). The resolution of the Hartman and Moosdorf map was 30 × 30 arc‐minutes, so each resulting global grid of mineral type was resampled using bilinear interpolation to 5 × 5 arc minutes, to match the other feature and observation grids. Additional information on the process is included in Appendices S1 and S2. Maps of the bicarbonate and calcium concentration of the GML output illustrate the typical global concentration distribution (Figures 2 and 3, Table 1; maps of other parameters are given in the Appendix S2).

**Table 1:** Predicted GML Global Mean and Median Concentration for Major, Selected Minor, and Trace Solutes, Plus Several Water Parameters for Active Aquifer Systems

Parameter	Ca	Mg	Na	K	Sr (μg/L)	Fe (μg/L)	HCO_3_	Cl	SO_4_
Mean	42	14	45	3.5	298	80	214	24	31
Stand. Dev	31	14	40	2.7	275	159	98	62	60
95%	200	194	802	25	2257	3322	522	2416	848
90%	190	184	760	23	2139	3147	494	2289	803
Median	106	103	423	13	1191	1749	276	1272	447
10%	22	21	85	2.8	244	350	57	255	90
5%	11	11	43	1.5	126	175	30	128	46

Parameter	NO_3_ as N	F	Br	Si	Dissolved oxygen	pH	Specific conductance μS/cm	Temperature (°C)
Mean	0.34	0.23	0.09	17	1.8	7.21	551	20.0
Stand. Dev	0.36	0.18	0.11	9	1.6	0.34	301	7.0
95%	9.0	1.6	1.1	46	7.8	8.28	1910	28.7
90%	8.5	1.5	1.1	43	7.4	8.09	1811	27.4
Median	4.8	0.84	0.60	24	4.2	6.57	1020	16.9
10%	0.96	0.18	0.13	5.4	0.9	5.04	229	6.4
5%	0.49	0.10	0.07	3.0	0.5	4.85	130	5.1

Note: Values are in mg/L except Fe and Sr (μg/L), pH (standard units), specific conductance (μS/cm), and temperature (°C).

## Discussion

The statistical data in Table 1 indicate the range of uncertainty by standard deviation about the mean and percentiles surrounding the median. The rather large standard deviation around the mean of some of the parameters is not unusual when it is recognized that over 2.1 million data cells are averaged. That is, the variation is largely the result of the full range of concentration differences over the entire global land surfaces and not analytical uncertainty.

The mean GML concentration of Cl^−^ (24 mg/L) and SO_4_^2−^ (31 mg/L) (Table 1) appear slightly greater than one might expect for relatively conservative solutes based on global rivers concentrations of 7.8 mg/L Cl^−^ and 11 mg/L SO_4_^2−^ (Meybeck 2003). That is, one might anticipate the global active aquifer values to be approximately twice the global river values based on the observation that approximately half the water in rivers is sourced from active aquifers (Reitz et al. 2017) and the other half from precipitation runoff; however, the Cl/SO_4_ ratios are similar in both the GML and global river water (0.7). The mean GML value of Na^+^ is significantly higher (45 mg/L) than might be expected based on global river water (5.2 mg/L; Meybeck 2003). Rapid flow from shallow flow paths in active aquifers will represent a larger proportion of river flow, and because solute concentrations generally increase with age (Appelo and Postma 2005), thus, the base flow has a bias toward younger groundwater. Additionally, as in the case with chloride and sulfate global riverine systems may not be representative of active aquifer systems in the 30% of the continents that are semi‐arid or arid that lack river systems and where the solutes in the active aquifer have higher concentrations.

The true mean global values of the major solute concentrations can never be known; however, the breadth of climatic, aquifer lithology, aquifer age, and other factors suggest that the GML concentrations summarized in Table 1 are representative of the true global values. To evaluate this assumption, we looked at several independent geochemical indicators. The chloride/bromide ratio is a potential check on the reasonableness of the mean GML values. That is, it is known that most of the chloride and bromide in the active aquifers are from ocean‐sourced precipitation (Davis et al. 1998). As the mean GML value of each solute is based on different predictors one can compare the known values with the predicted values. The GML chloride to bromide ratio is 240 (Table 1) and the ocean ratio is approximately 285 (Davis et al. 1998). Thus, we conclude that the GML values are consistent with the true values. An additional check on the representativeness of our GML values is shown by thermodynamic equilibrium calculation. Most individual analyses of water from active aquifer systems are close to equilibrium with the mineral calcite thus, if the calculated GML values of Ca^2+^, HCO_3_^−^, pH, and temperature are representative, the GML values should yield an equilibrium calculation close to equilibrium. Using the geochemical code PHREEQC (Parkhurst and Appelo 2013) and the global values from Table 1 documents that the solute concentration from the global active aquifers is slightly undersaturated with respect to calcite, thus consistent with general observations (Appendix S1). Finally, the cation/anion balance error of major ions (Table 1) is less than 5% (+3.4), even though each solute was generated by different predictors. These evaluations are consistent with the mean predicted values being reasonable representations of true solute concentration and ionic composition of water from active freshwater aquifers.

In our analyses we have assumed steady‐state solute conditions; however, it is clear that the solute composition and concentration of active groundwater systems are changing with time in response to both global climate change and anthropogenic activity. Thus, the values presented represent a snapshot in time. Further, nearly all the solute samples were analyzed within the last 20 years and thus, do not represent geogenic conditions. This condition is no different from the global surface water composition that is changing with time for the same reasons. We suspect that most anthropogenic changes will certainly affect local aquifers; however, it is more likely atmospheric introduction of contaminates like bomb tritium or chlorofluorocarbons (CFC) will impact the active groundwater system on a global scale. The actual changes in solute concentration and composition that occur will depend largely on the source of the solutes in the aquifers (precipitation, transport from adjacent aquifers, legacy/connate, or rock‐water interaction).

We suspect that the model could be further improved with additional predictor datasets. Ideally, global estimates of discharged weighted values of concentration for every aquifer and “aquifer reactivity” (essentially the number of aquifer pore‐volumes discharged since flow in the aquifer became active) would improve solute characterization. However, neither predictor database currently exists and the data input requirements for them were beyond the scope of our project.

## Conclusions

Our GML prediction of groundwater solute concentration (Table 1) provides a composition and concentration of global groundwater. It is hoped that these values will help inform future continental‐scale mass‐flux calculations of critical elements such as carbon for climate change; nutrients such as nitrogen for eutrophication and legacy management; contribution of groundwater to continental denudation; and understanding of groundwater weathering and geochemical cycling of the elements. The uncertainty of the values is given both by standard deviation of the mean and percentiles surrounding the median. In addition to global solute fluxes and element cycling, the concentrations might be used to compare with data from local aquifers to gain some insight into the local reactions controlling the solute in an aquifer. It might also be used when plotting on geochemical graphical displays (Stiff, Pie, Piper, Scholler, Durov, probability distribution. etc.) to illustrate the deviation of a local aquifer from that of the global value.

## Author Contributions

W.W.W. and J.A.C. recognized the need for the global active aquifer concentration values, wrote a draft outline of the project, and recruited the other participants. P.L.S. curated and supervised the solute database and provided statistical data from the UK baseline project. B.D.L. provided USGS solute data, a map of Australian data locations, and geologic geospatial predictors working with W.T.W. who performed the GML approach and calculated the global concentration values. R.E.K. provided the Brazilian database and geochemical insight. All authors contributed to the final writing and editing.

### Authors' Note

The authors do not have any conflicts of interest or financial disclosures to report.

## Supporting information


**Appendix S1:** Maps of solute data locations; statistical analysis of solute data set (SM1‐1 Table 1); statistical analyses of solute database versus random database (SI‐1 Table 2); thermodynamic analyses of the GML predictions and details on the geospatial machine learning algorithm.

**Appendix S2:** Global maps of groundwater concentrations of solutes and chemical parameters.

**Appendix S3:** Table of complete solute database used in GML algorithm—latitude and longitude in decimal degrees, solutes in mg/L except Sr and Fe (μg/L), pH in pH units, specific conductance in micro‐Siemens (μS), and temperature in degrees Centigrade.
