# Supplementary material for: Global Groundwater Solute Composition and Concentrations
Source: Ground Water. 2022 May 26;60(6):714–20. doi: 10.1111/gwat.13205 (PMC9796178; doi:10.1111/gwat.13205)
Supplement: Supplementary file 1 — Appendix S1: Maps of solute data locations; statistical analysis of solute data set (SM1‐1 Table 1); statistical analyses of solute database versus random database (SI‐1 Table 2); thermodynamic analyses of the GML predictions and details on the geospatial machine learning algorithm. [file GWAT-60-714-s001.docx]

**Global Groundwater Solute Composition and Concentrations**

Warren W. Wood^1^, Pauline L. Smedley^2^, Bruce D. Lindsey^3^, Warren T. Wood^4^, Roberto E. Kirchheim^5^, and John A. Cherry^6^

**Supporting Information**

**Appendix 1**

**Solute database**

The solute database was compiled from a combination of the Australian Meteorological Survey Global Explorer, Geological Survey of Brazil, Geological Survey of Chile, and the U. S. Geological Survey. See SI-Figs 1 through SI-4 for distribution of the samples.

**
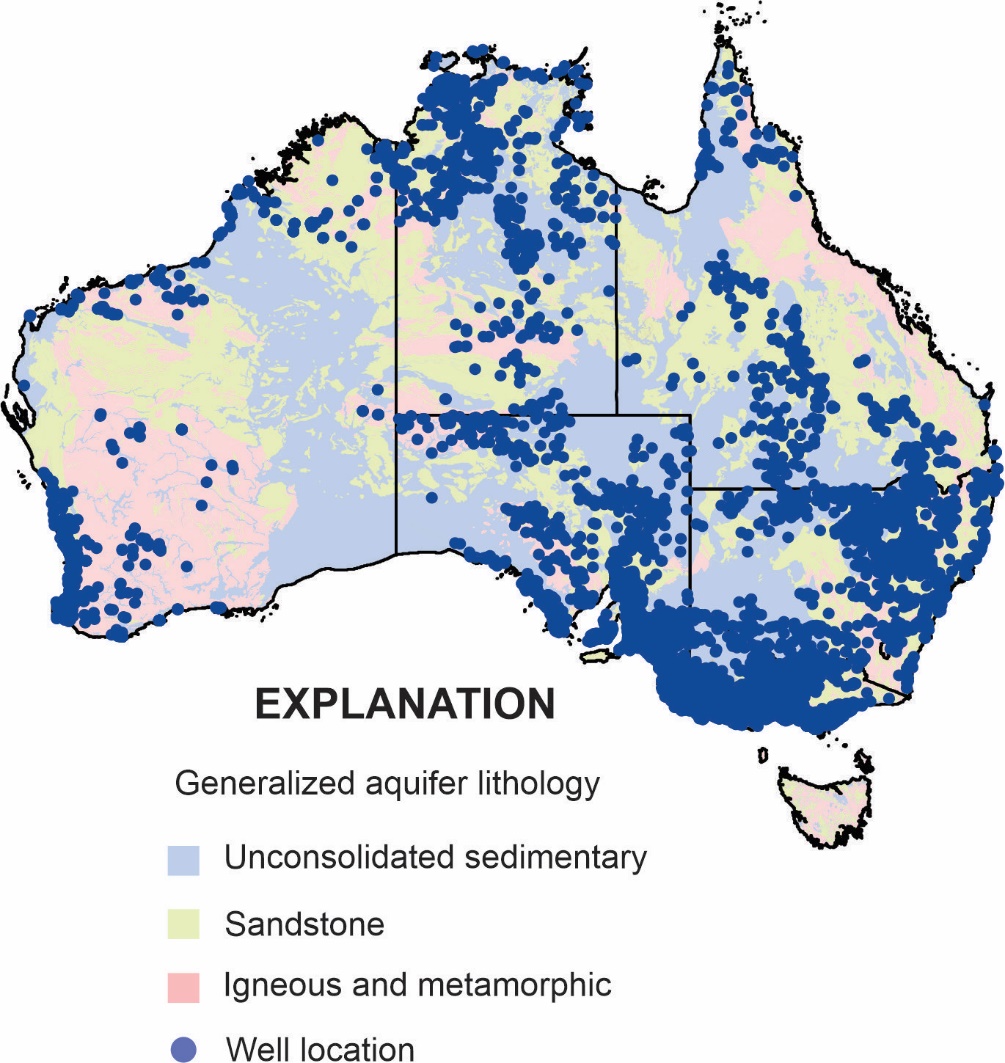
**

SI-1 Figure 1 -- Location of the sampling points of groundwater from aquifers in the “Groundwater Explorer” database, Australian Bureau of Meteorology. (Basemap from Raymond et al., 2012).

SI-1 Figure 2 -- Location of groundwater sampling points from aquifers in Brazil. Geological Survey of Brazil (Serviço Geológico do Brasil) SIAGAS “Groundwater Information System”.

**-**
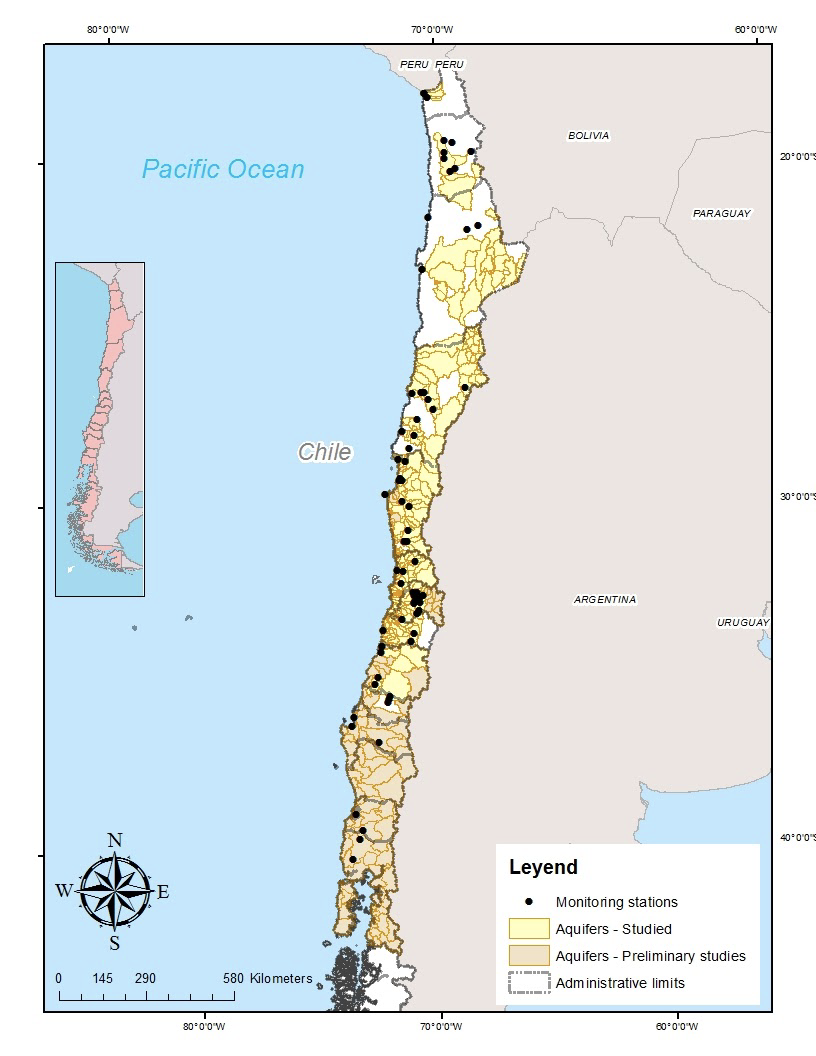


SI-1 Figure 3 -- Locations of groundwater sampling points from Banco Nacional de Aguas, Republic of Chile.

**
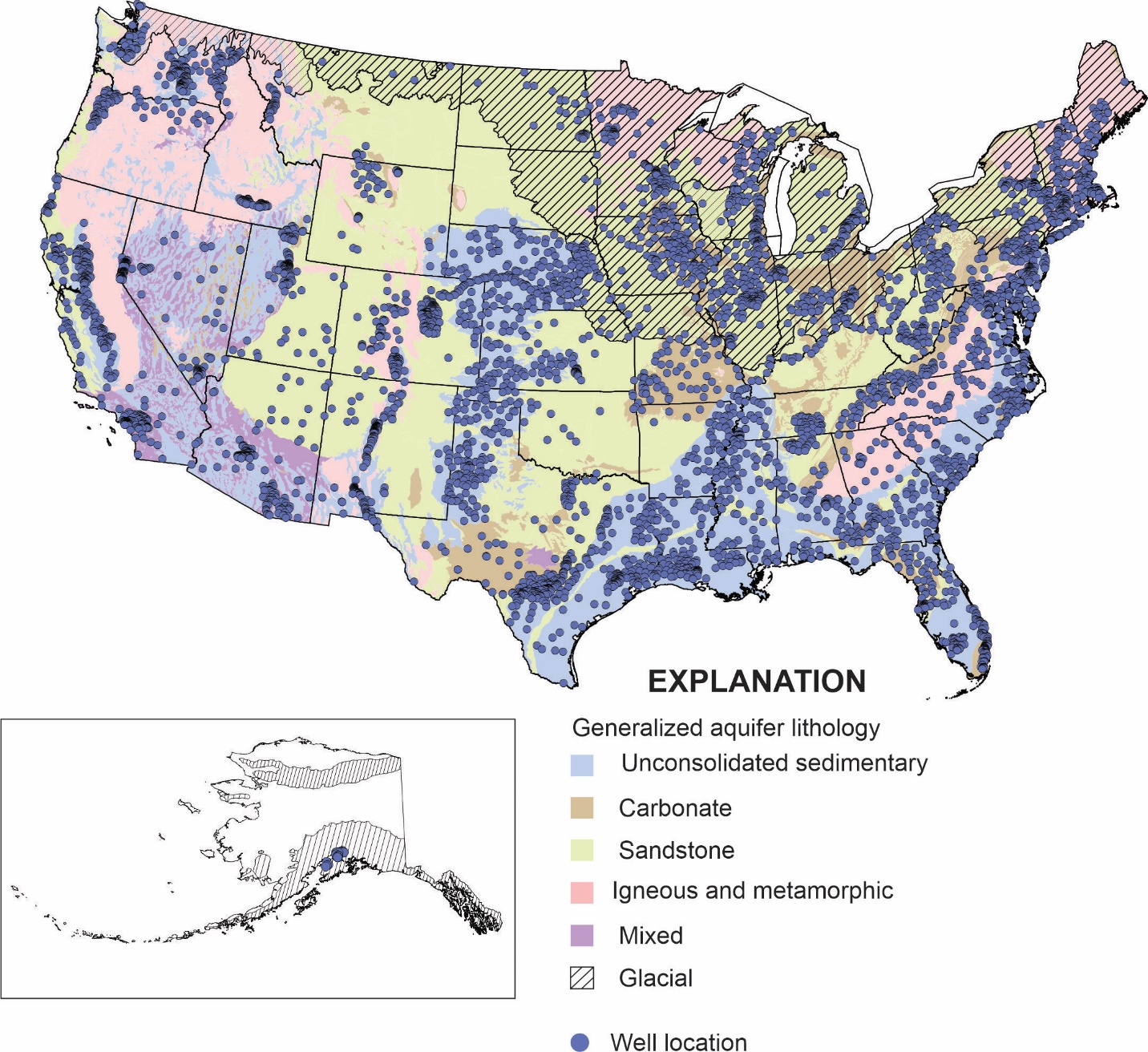
**

SI-1 Figure 4 -- Location of the sampling points of groundwater from aquifers in the United States. U.S. Geological Survey’s NAWQA (National Water Quality Assessment) (Base map: U.S. Geological Survey, 2003; Belitz et al., 2019).

Owing to differences in laboratory procedures, evolving analytical methods, and agency goals, different approaches have been used in reporting of “non-detects” or “below detection” for minor or trace elements in different databases (Helsel, 2005). Some values are reported as ND “non-detect”; use a dash (--) assign 0.00 mg/L, use the word “zero” or other limiting value; -0.02 was used to flag these values in our database. The statistical summary of this database in given in SI Table 1 and the full data set is provided in SI -3

**SI-1 Table 1 -- Statistical summary of solute database of active aquifer systems**

**SI-1 Table 2- Statistical analyses bicarbonate groundwater data from outside our database area**

**(**UK and China data are unpublished)

Previously published concentration and statistical analyses from areas not in our database (Algeria, Canada, China, Costa Rico, Egypt, India, Mexico, South Africa, and United Kingdom) are presented in SI-1 Table 2 Owing to the general normal distribution of bicarbonate concentration, mean and median are similar and the median value was substituted calculating a sample weighted mean value [ Σ(Ni x Ci)/Σ Ni] of 242 mg/L bicarbonate, where N is number of samples and C is concentration (SI-1 Table 2)

**Thermodynamic evaluation of the GML estimates**

Thermodynamic calculation for calcite saturation for most groundwaters are near equilibrium. To test if our proposed values are consistent with this operation we utilized our calculated geospatial machine learning (GML) values of pH, bicarbonate, calcium, and temperature with the code PHREEQC (Parkhurst and Appelo, 2013) to test if the calculated values are near equilibrium (Table 4-SM). The result of this calculation is consistent with the proposal that the GML values for parameters pH, temperature, calcium, and bicarbonate are representative of real system values. This calculation was made with the following input file.

SOLUTION 1

-units mg/L

pH 7.2

Temp 20.0

Ca 42

Alkalinity 214 as HCO3

END

**SI-1 Table 4 -- PHREEQC Output of Solution 1, SI = saturation indices, IAP = Ion Activity Product, and K is the equilibrium constant, Log is logarithm to base 10.**

| Phase | SI | Log IAP | Log K | Formula |
| --- | --- | --- | --- | --- |
| Aragonite | -0.20 | -8.54 | -8.34 | CaCO_3_ |
| Calcite | -0.06 | -8.54 | -8.48 | CaCO_3_ |
| CO_2_(g) | -2.06 | -3.53 | -1.47 | CO_2_ |
| H_2_ (g) | -22.83 | -25.93 | -3.10 | H_2_ |

**Geospatial machine learning**

Our objective in this analysis was to mitigate potential bias in our data sets of solute samples that could be caused by disproportionate sampling of disparate geochemical and hydrologic environments. Our available samples are very unevenly distributed over the Earth’s land area thus, the use a geospatial interpolation technique to predict solute concentration everywhere globally, even where they have not been observed, to simulate a regular sampling of the earth. For our single global average concentration, we integrate over the predicted solute grid weighted by the area of each cell. This weighted integration of the prediction mitigates bias in both the geographic and geologic distribution of the sparse samples.

The interpolation we employ is geospatial machine learning (GML). Our method employs the well-established k nearest neighbor (kNN) technique of Fix and Hodges (1951), although we expect similar results from other ML techniques that relay on correlations between data and predictors. The GML used here is described in Lee et al. (2019), to predict seafloor organic carbon concentration and is modified for the application to groundwater solute concentrations. The overall GML we are using has been demonstrated on a variety of geologic parameters, and recently published in the marine geology and geophysics literature (e.g., Martin, et al., 2015; Lee et al., 2019; Phrampus et al., 2020; Obelcz et al., 2020; Restreppo et al. 2020; Lee et al., 2020; Graw et al., 2020; Eymold et al., 2021; Restreppo et al., 2021)

Fundamentally, GML finds correlations between sparsely sampled observed values and aspects or features of the environment we know (or can accurately estimate) everywhere. The features are gridded values of quantities that represent what we know about the environment - topography, sediment type, rock type, climate, etc. GML then uses these correlations to make predictions of observed values where no values were directly observed; we predict what we would observe if we could. The overarching assumption is that there are one or more features of the environment that will correlate with a given observed global quantity, such as solute concentration.

There are several steps to the GML process, observation gridding, feature generation, feature selection, validation, and prediction. In our implementation of GML, the observations and features must both exist and be registered to the same grid. In general, the GML is agnostic to grid orientation or cell size, but for this analysis we chose a cell (pixel) centered grid of 5 x 5 arc-minutes in latitude and longitude (about 10 x 10 km at the equator). After gridding, cells containing three or more observed values are represented by the median of those values, cells containing exactly two observed values are represented by the mean of those two values, and cells with a single observed value are represented by that value. Cells with no observed values are the cells for which predicted values are generated.

Features of the environment must have values at all cells where a prediction is to be made – all cells on land in our case. The features in the analysis come from several publicly available sources. Elevation was obtained from SRTM15+ (Tozer et. al, 2020). The topographic value at each cell was taken to be the median of the 20 x 20 15 arc-second cells in each 5 arc-minute cell. Climate data was obtained from the World climate database (2021) and included annual averages, minima, maxima, and standard deviations of temperature, precipitation, solar radiation, wind speed and water vapor pressure, all at 5x5 arc minutes. Another set of features was generated from a digital geological map produced by Hartmann and Moosdorf (2012). The provinces in this map were descriptive, so to be useful for our analysis each of the thirteen rock types was converted to fraction of dominant mineral types (silica, calcium, iron, and potassium). The resolution of the Hartman and Moosdorf map was 30 x 30 arc minutes, so each resulting global grid of mineral type was resampled using bilinear interpolation to 5 x 5 arc minutes, to match the other feature and observation grids. Maps of the major solute and other parameters of the machine learning output are given in SI-3.

**References cited in the Supporting Information**

Adamas, S., Titusa, R., Pietersen, K. Tredouxc, G. Harris, C., 2001, Hydrochemical characteristics of aquifers near Sutherland in the Western Karoo, South Africa; J. of Hydrology v. 241, p. 91–103.

Belitz, K., Watson, E., Johnson, T.D., and Sharpe, J., 2018, Identification of Secondary Hydrogeologic Regions in areas outside of Principal Aquifers expands the framework for U.S. national assessments; Groundwater, v. 57, p. 367-377. https://doi.org/10.1111/gwat.12806

Belkhiri, L., Boudoukha, A., and Mouni, L.P., 2011, A multivariate Statistical Analysis of Groundwater Chemistry Data; Int. J. Environ. Res., v. 5, p. 537-544.

Bondu, R., Cloutier, V., Rosa, E., and Roy, M., 2020, An exploratory data analysis approach for assessing the sources and distribution of naturally occurring contaminants (F, Ba, Mn, As) in groundwater from southern Quebec (Canada); Applied Geochemistry, v. 114, 17 p.

DeSimone, L. A., McMahon, P. B., and Rosen, M. R., 2014, The quality of our Nation’s waters — Water quality in Principal Aquifers of the United States, 1991–2010; U.S. Geological Survey Circular 1360, 151 p., https://dx.doi.org/10.3133/cir1360.

Eymold, W. K., Frederick, J. M., Nole, M., Phrampus, B. J., and Wood, W. T., 2021, Prediction of gas hydrate formation at Blake Ridge using machine learning and probabilistic reservoir simulation; Geochemistry, Geophysics, Geosystems, v. 22, 22 p. e2020GC009574. <https://doi.org/10.1029/2020GC009574>

Fix, E. and Hodges, J. L. 1951, Discriminatory Analysis. Nonparametric Discrimination: Consistency Properties; Report Number 4, Project Number 21-49-004, USAF School of Aviation Medicine, Randolph Field, Texas,

Graw, J. H., Wood, W. T., and Phrampus, B. J., 2020, Predicting global marine sediment density using the random forest regressor machine learning algorithm; J. of Geophysical Research: Solid Earth, v. 126, e2020JB020135. <https://doi.org/10.1029/2020JB020135>

Hartmann, J., and N. Moosdorf, 2012, The new global lithological map database GLiM: A representation of rock properties at the Earth surface; Geochem. Geophys. Geosyst., v. 13, 37 p. doi:10.1029/2012GC004370.

Helsel, D., R., 2005. Non-detects and Data Analysis: Statistics for Censored Environmental Data. John Wiley, New York.  268 p.

Lee, T. R., Phrampus, B. J., Obelcz, J., Wood, W. T., and Skarke, A, 2020, Global Marine Isochore Estimates Using Machine Learning; Geophysical Research Letters, v. 21, 9 p. <https://doi.org/10.1029/2020GL088726>

Lee, T. R., Wood, W. T., & Phrampus, B. J., 2019, A machine learning (kNN) approach to predicting global seafloor total organic carbon; Global Biogeochemical Cycles, 33. p. 37-46 <https://doi.org/10.1029/2018GB005992>

Mahlknecht, J., Steinich,. B. and Navarro de Leon, I., 2004, Groundwater chemistry and mass transfers in the Independence aquifer, central Mexico, by using multivariate statistics and mass-balance models; Environmental Geology v. 45, p. 781–795. DOI 10.1007/s00254-003-0938-3

Martin, K. M., Wood, W. T. and Becker, J. J., 2015, A global prediction of seafloor sediment porosity using machine learning; Geophys. Res. Lett, v. 42, p. 10,640-10,646 DOI: 10.1002/2015GL065279

Masoud, A. A., 2014, Groundwater quality assessment of the shallow aquifers west of the Nile Delta (Egypt) using multivariate statistical and geostatistical techniques; Journal of African Earth Sciences, v. 95, p 123–137

Obelcz, J., Wood, W. T., Phrampus, B. J., and Lee, T. R., 2020, Machine learning augmented time‐lapse bathymetric surveys: A case study from the Mississippi river delta front; Geophysical Research Letters, v. 47, 8 p. e2020GL087857. <https://doi.org/10.1029/2020GL087857>

Parkhurst, D. L., and Appelo, C. A. J., 2013, Description of input and examples for PHREEQC version 3—A computer program for speciation, batch-reaction, one-dimensional transport, and inverse geochemical calculations; U.S. Geological Survey Techniques and Methods, Book 6, chap. A43, 497 p.

Personal Communication, Pauline L Smedley, 2021, British Geological Survey for statistical data on groundwater bicarbonate values in the United Kingdom

Personal Communication, Wenpeng Li and. Longfeng Wang, 2021, China Institute of Geo-Environmental Monitoring for statistical data on groundwater bicarbonate values in China.

Phrampus, B. J., Lee, T. R., and Wood, W.T., 2020, A global probabilistic prediction of cold seeps and associated SEAfloor FLuid Expulsion Anomalies (SEAFLEAs); Geochemistry, Geophysics, Geosystems, v. 21 16 p. e2019GC008747. <https://doi.org/10.1029/2019GC008747>

Raymond, O. L., Gallagher, R., and Highlet, L. M., 2012, Surface Geology of Australia, 1:2,500,000 scale; 2012 edition; Commonwealth of Australia (Geoscience Australia)

Redwan, M., Abdel Moneim, A. A., 2016, Factors controlling groundwater hydrogeochemistry in the area west of Tahta, Sohag, Upper Egypt; Journal of African Earth Sciences, v. 118, p. 328-338

Restreppo, G. A., Wood, W. T. and Phrampus, B. J., 2020, Oceanic sediment accumulation rates predicted via machine learning algorithm: towards sediment characterization on a global scale; Geo-Mar Lett. v. 40, p. 755–763. <https://doi.org/10.1007/s00367-020-00669-1>

Restreppo, G. A., Wood, W. T., J Graw, J. H., and Phrampus, B. J., 2021, A machine-learning derived model of seafloor sediment accumulation; Marine Geology, v 440, 6 p. 106577, <https://doi.org/10.1016/j.margeo.2021.106577>

Sanchez-Gutierrez, R., Mena-Rivera, L., Sanchez-Murillo, R., Fonseca-Sanchez, A., and Madrigal-Solıs, H., 2020, Hydrogeochemical baseline in a human-altered landscape of the central Pacific coast of Costa Rica; Environ Geochem Health v. 42, p. 2685-2701. https://doi.org/10.1007/s10653-019-00501-5(

Singh, K. P., Malik, A., Vinod K. Singh, V. K., Mohana, D., and Sinha, S., 2005, Chemometric analysis of groundwater quality data of alluvial aquifer of Gangetic plain, North India; Analytica Chimica Acta v. 550, p. 82–91.

Tozer, B. , D. T. Sandwell, W. H. F. Smith, C. Olson, J. R. Beale, and P. Wessel, 2019, Global bathymetry and topography at 15 arc seconds: SRTM15+; [Earth and Space Science](https://agupubs.onlinelibrary.wiley.com/journal/23335084), v. 6, p. 1847-1864.  <https://doi.org/10.1029/2019EA000658>

U.S. Geological Survey, 2003, Principal Aquifers of the 48 conterminous United States, Hawaii, Puerto Rico, and the U.S. Virgin Islands: U.S. Geological Survey National Atlas of the United States Web site. <https://water.usgs.gov/ogw/aquifer/map.html>

Wendland, F., Hannappel, S., Kunkel, R., Schenk, R., Voigt, H.J. and Wolter, R. A., 2003, Procedure to define natural groundwater conditions of groundwater bodies in Germany; Diffuse Pollution Conference Dublin, p. 7-20 to 7-25

**Author address and contact information**

^1^Warren W. Wood,

Visiting Professor, Department of Earth and Environmental Sciences,

206 Natural Sciences Building,

288 Farm Lane,

Michigan State University,

East Lansing, Michigan 48824

USA

<[wwwood@msu.edu](mailto:wwwood@msu.edu)> corresponding author

^2^Pauline L. Smedley,

Hydrogeochemist,

British Geological Survey.

Nicker Hill,

Keyworth.

Nottingham, NG12 5GG,

UNITED KINGDOM

^3^Bruce D. Lindsey,

Groundwater Status and Trends Coordinator

U.S. Geological Survey

215 Limekiln Road

New Cumberland, PA 17070

USA

^4^Warren T. Wood

Geophysicist. Head, Geology and Geophysics Section

Naval Research Laboratory,

NRL Code 7432

John C. Stennis Space Center, MS 39529

USA

^5^Roberto E. Kirchheim –

National Coordinator of the Program for Isotope and Hydrochemistry

Geological Survey of Brazil (CPRM‐SGB)

Rua Costa, 55 - Cerqueira César
São Paulo - SP - Brazil
CEP: 01304-010

BRAZIL

^6^John A. Cherry,

Professor and Associate Director of Morwick G360 Institute for Groundwater Research,

University of Guelph,

50 Stone Road East, Thornbrough Building,

Guelph, Ontario, N1G 2W1

CANADA
