# Supplementary material for: Global Groundwater Solute Composition and Concentrations
Source: Ground Water. 2022 May 26;60(6):714–20. doi: 10.1111/gwat.13205 (PMC9796178; doi:10.1111/gwat.13205)
Supplement: Supplementary file 2 — Appendix S2: Global maps of groundwater concentrations of solutes and chemical parameters. [file GWAT-60-714-s003.docx]

**Global Groundwater Solutes Composition and Concentrations**

Warren W. Wood^1^, Pauline L. Smedley^2^, Bruce D. Lindsey^3^, Warren T. Wood^4^, Roberto E. Kirchheim^5^, and John A. Cherry^6^

**Supporting Information**

**Appendix 2**

Maps of global concentration of solutes and water parameters


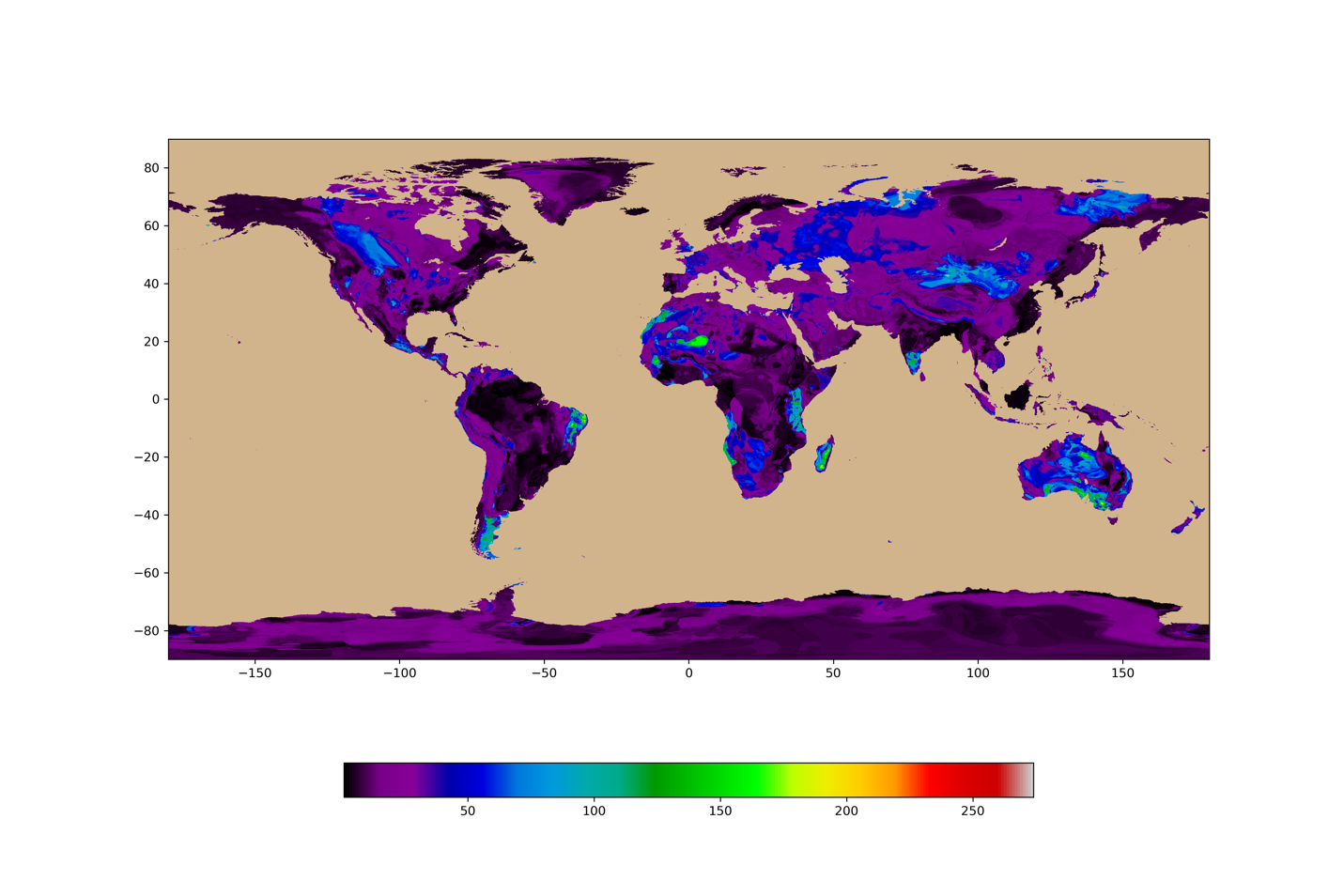


SI-2 Figure 1-- GML map of groundwater magnesium concentration, in mg/L.


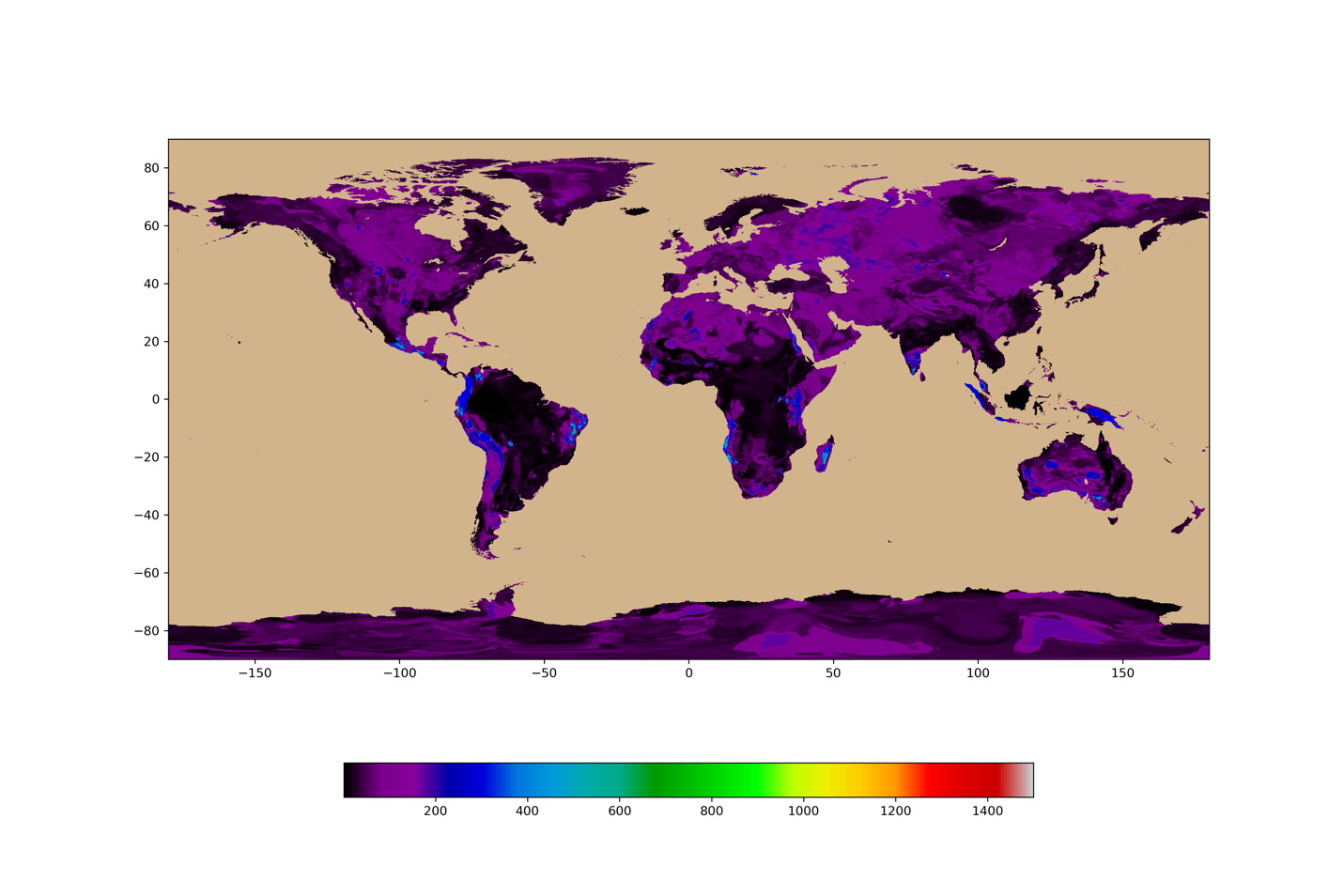


SI-2 Figure 2 -- GML map of groundwater sodium concentration, in mg/L.


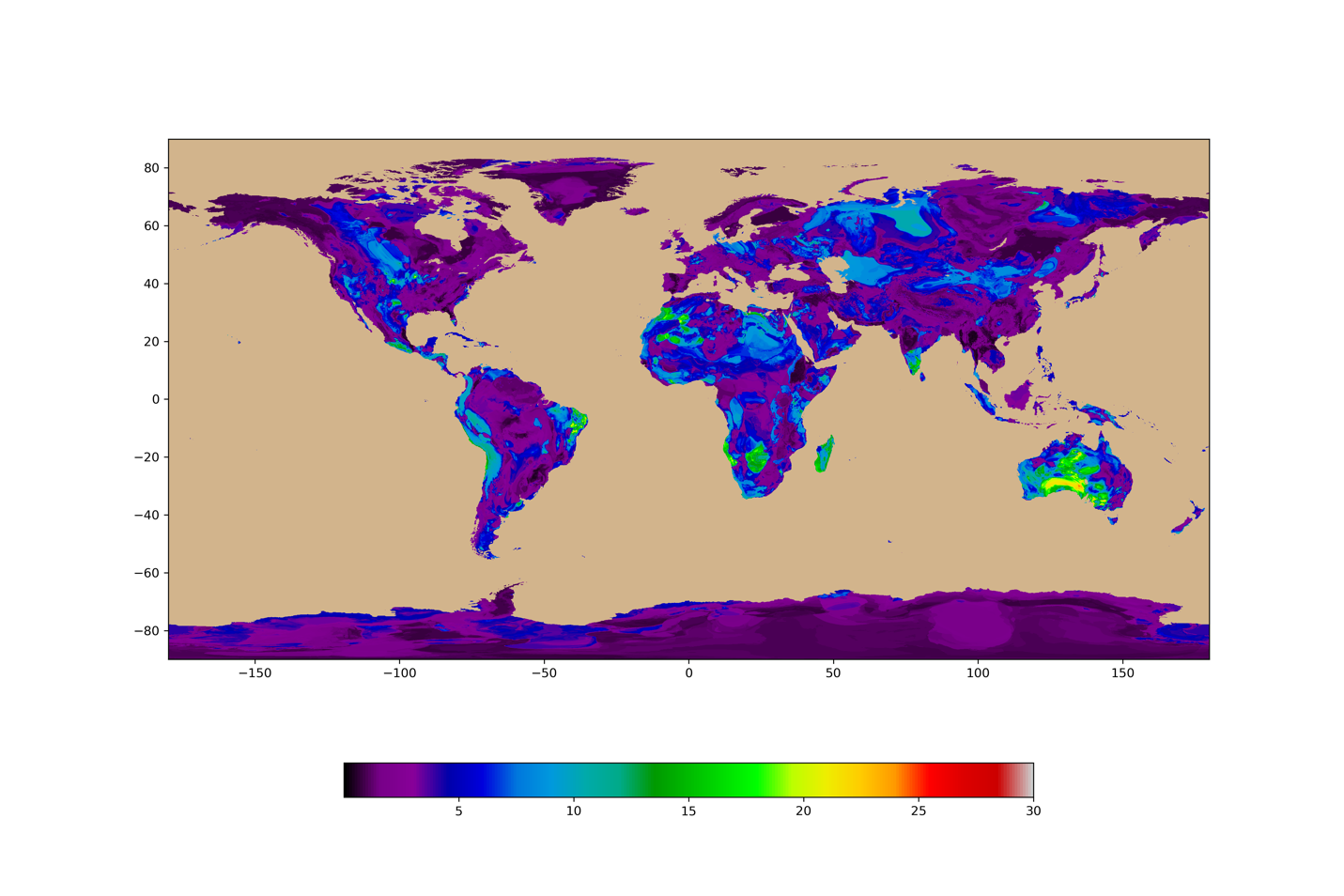


SI-2 Figure 3 -- GML map of groundwater potassium concentration, in mg/L.


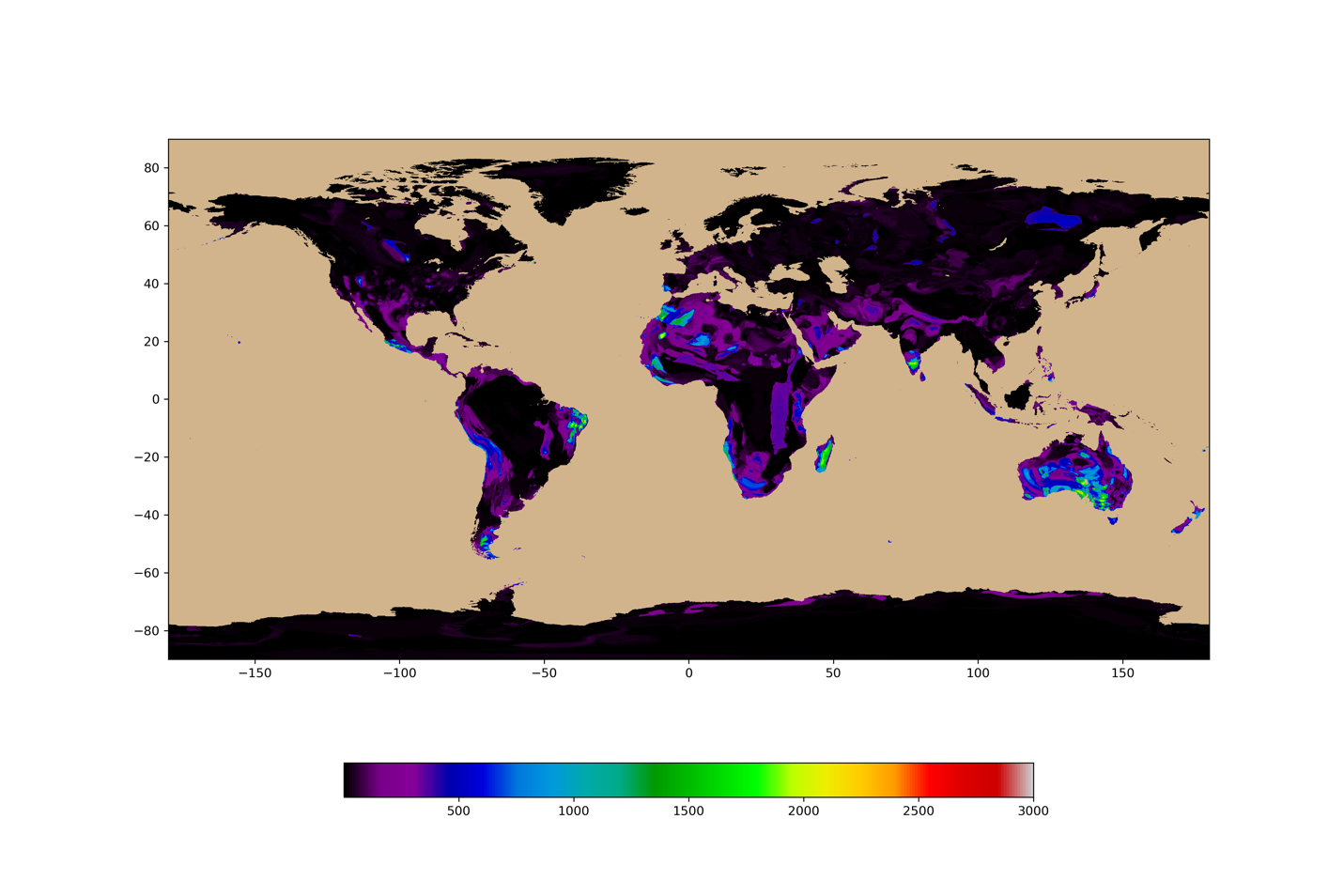


SI-2 Figure 4 -- GML map of groundwater chloride concentration, in mg/L.


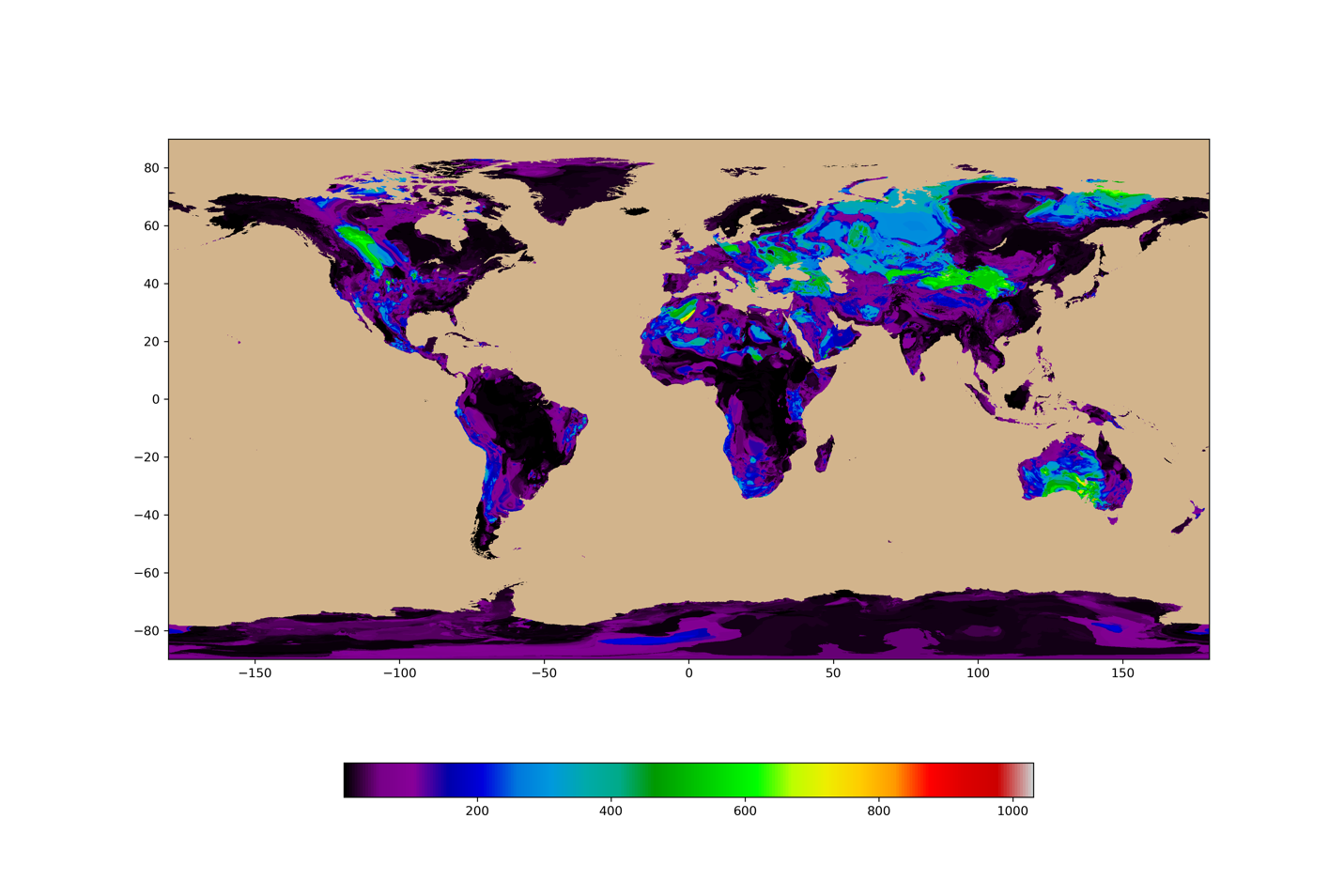


SI-2 Figure 5 -- GML map of groundwater sulfate concentration, in mg/L.


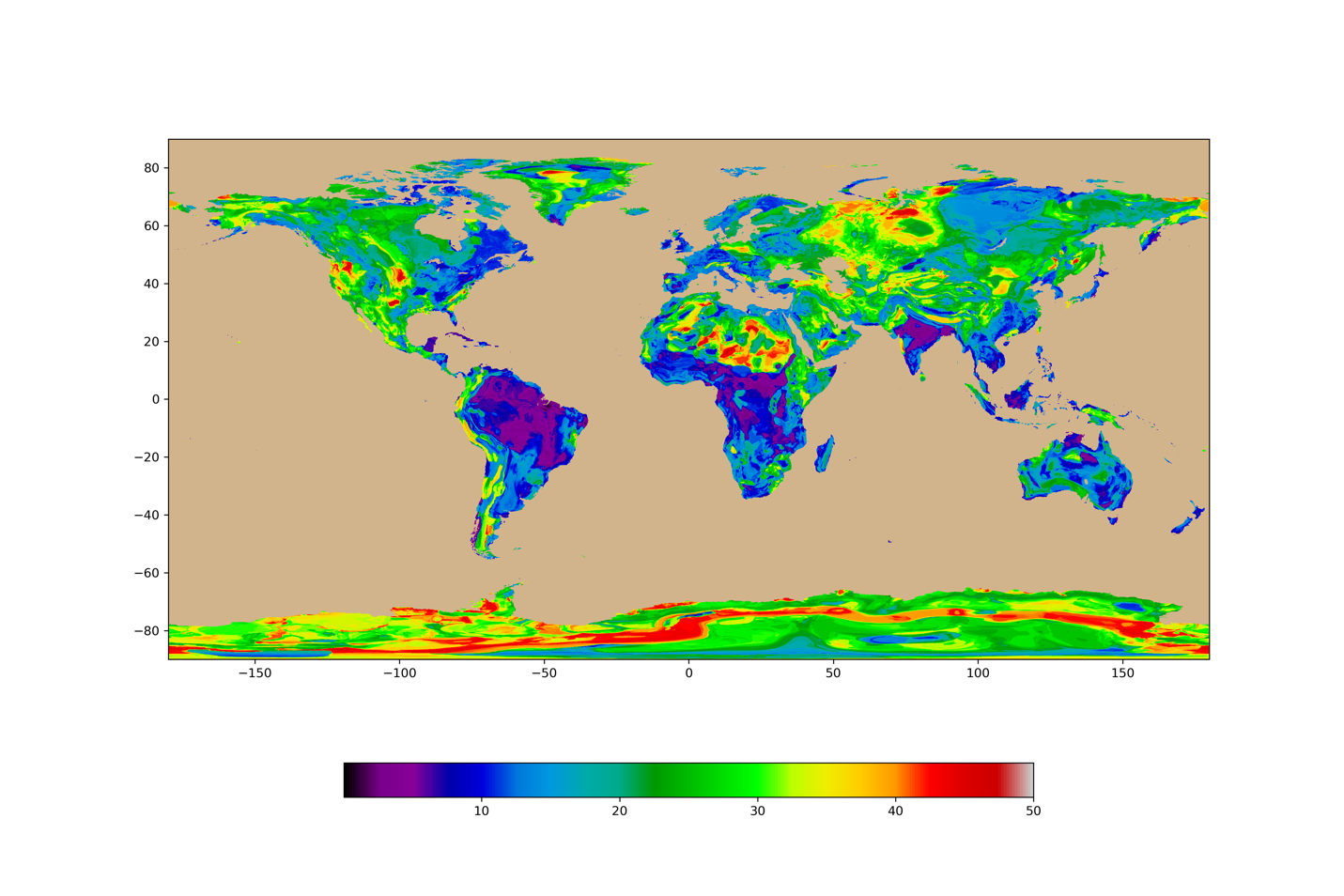


SI-2 Figure 6 -- GML map of groundwater silica (Si), in mg/L


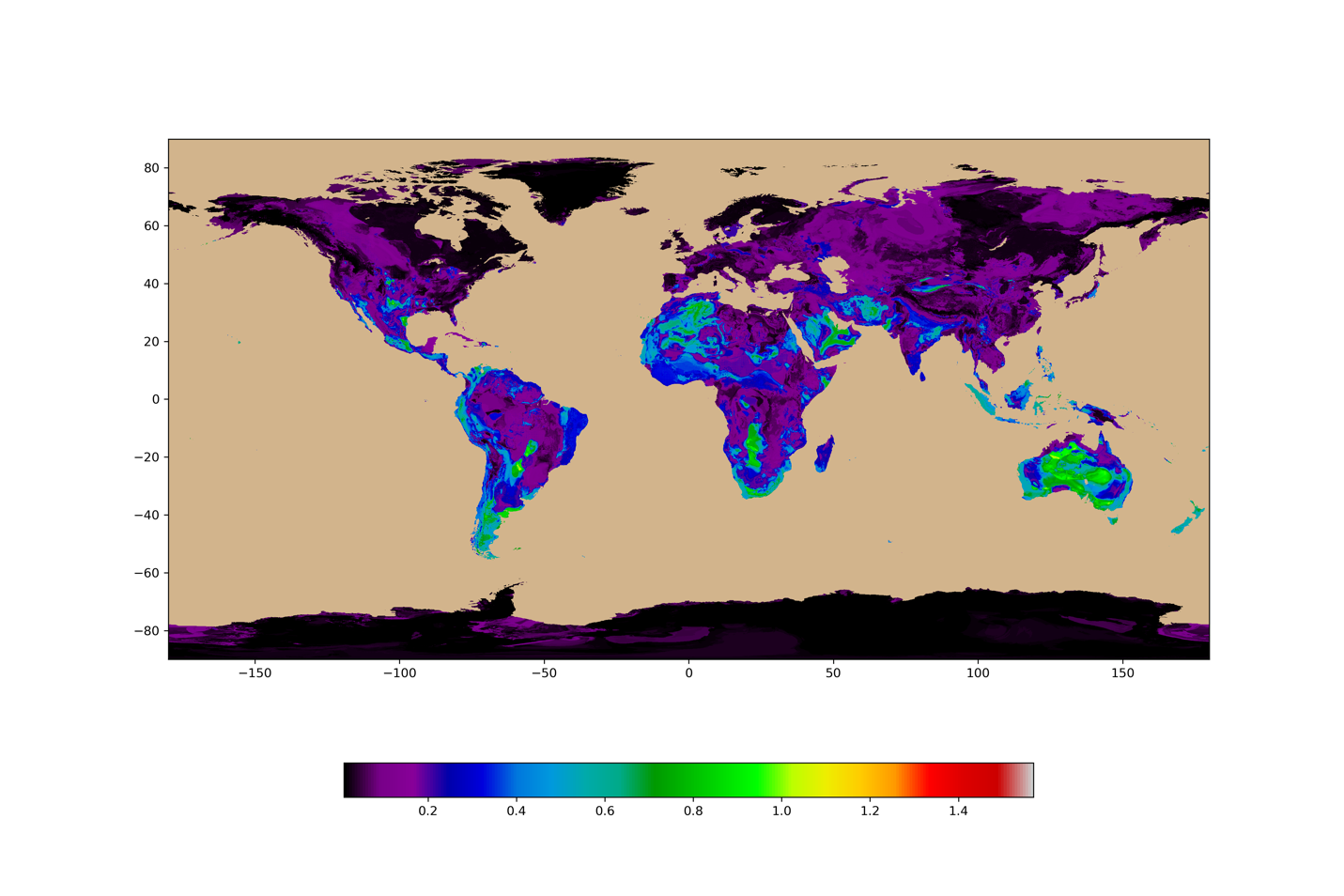


SI-2 Figure 7 -- GML map of groundwater bromide, in mg/L


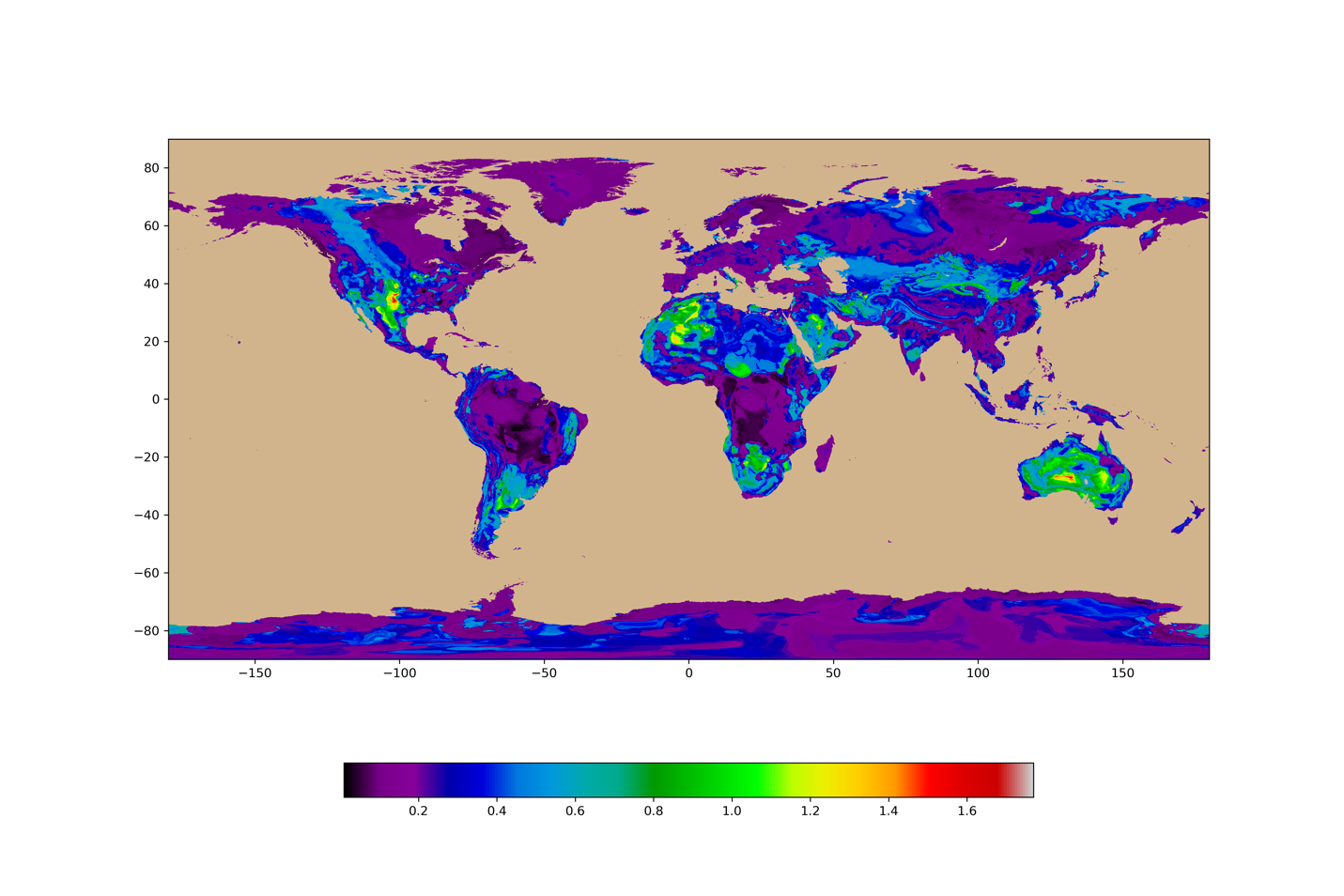


SI-2 Figure 8 -- GML map of groundwater fluoride, mg/L


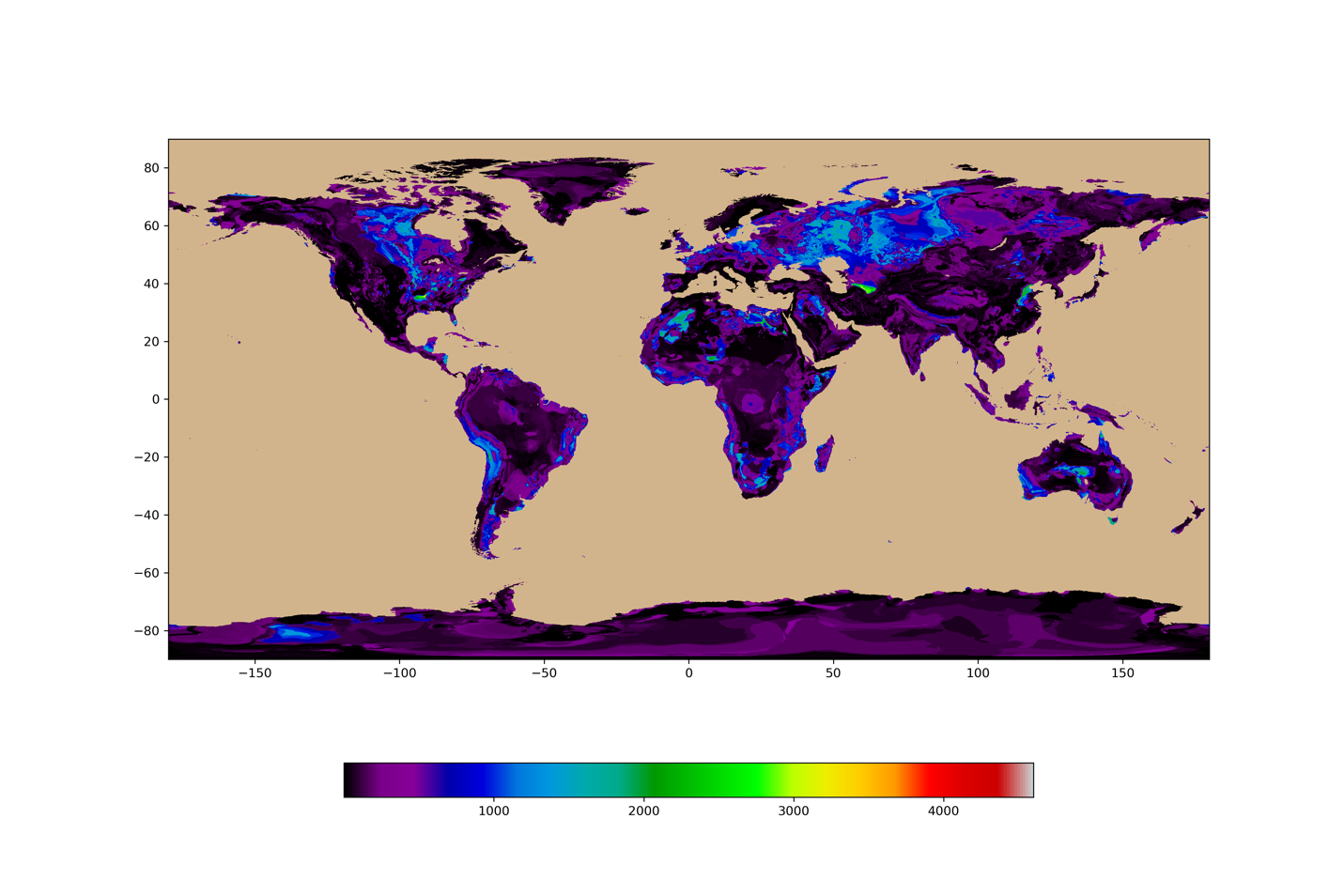


SI-2 Figure 9 -- GML map of groundwater iron, in ug/L.


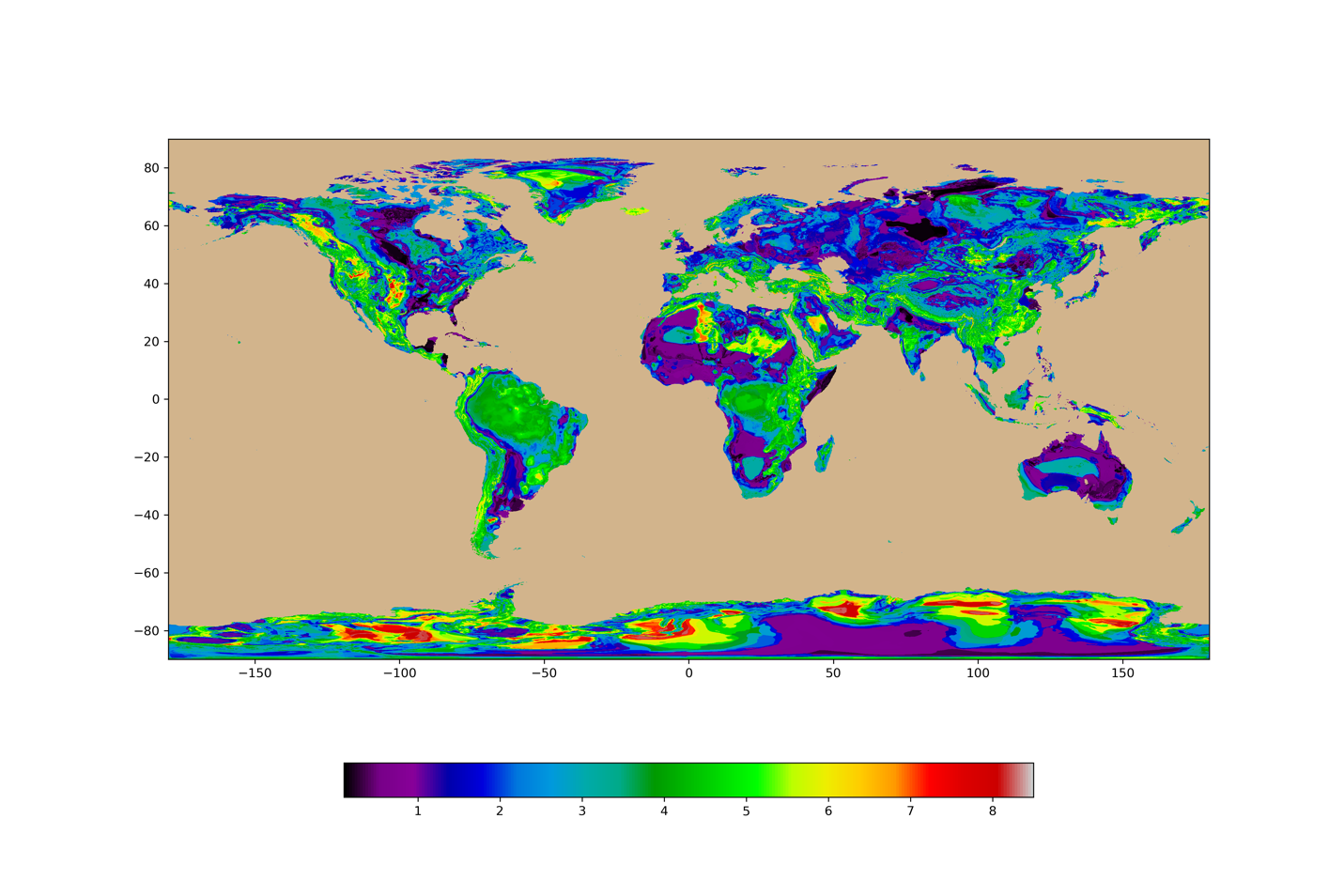


SI-2 Figure 10 -- GML map of groundwater of dissolved oxygen, in mg/L


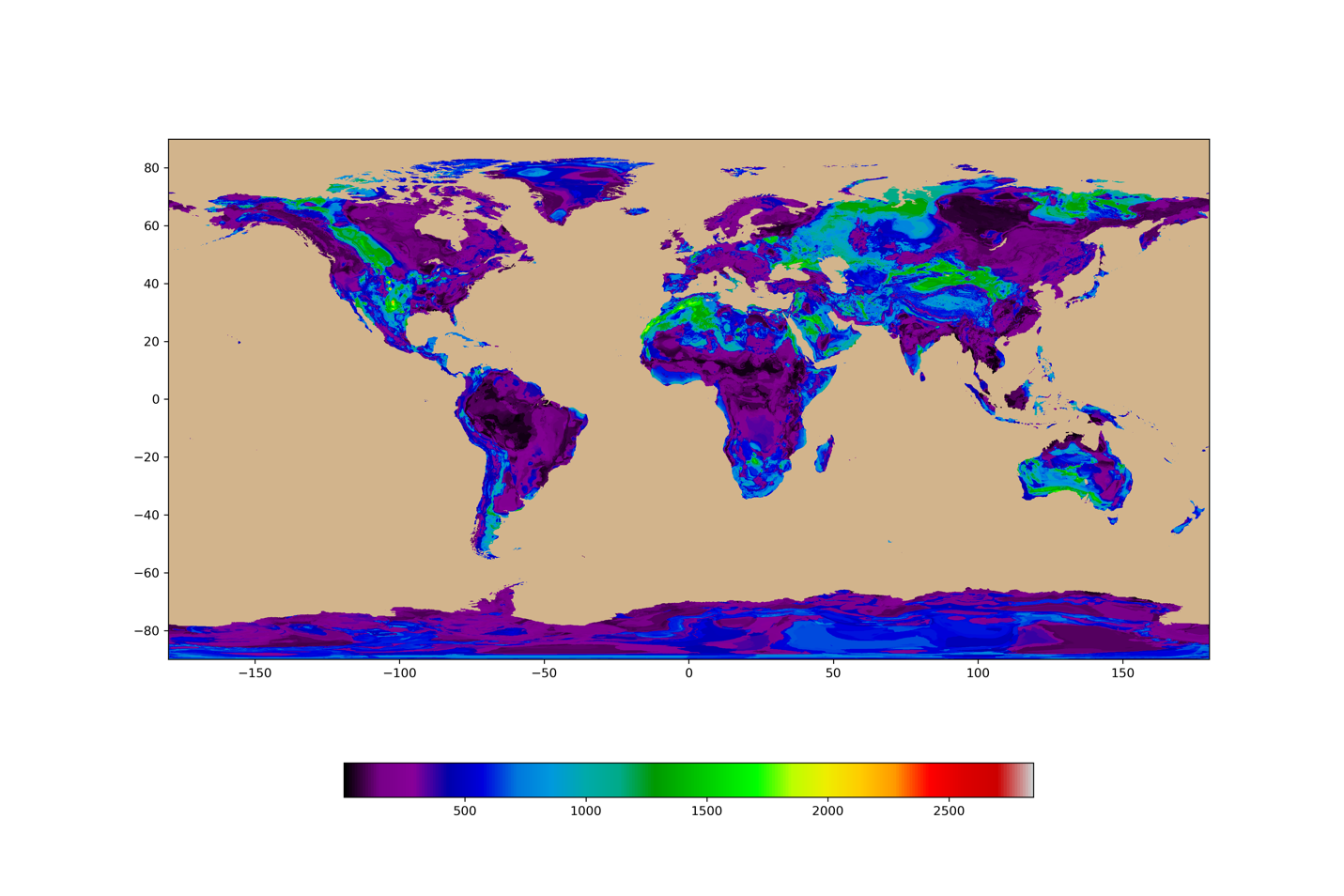


SI-2 Figure 11 -- GML map of groundwater strontium, in ug/L


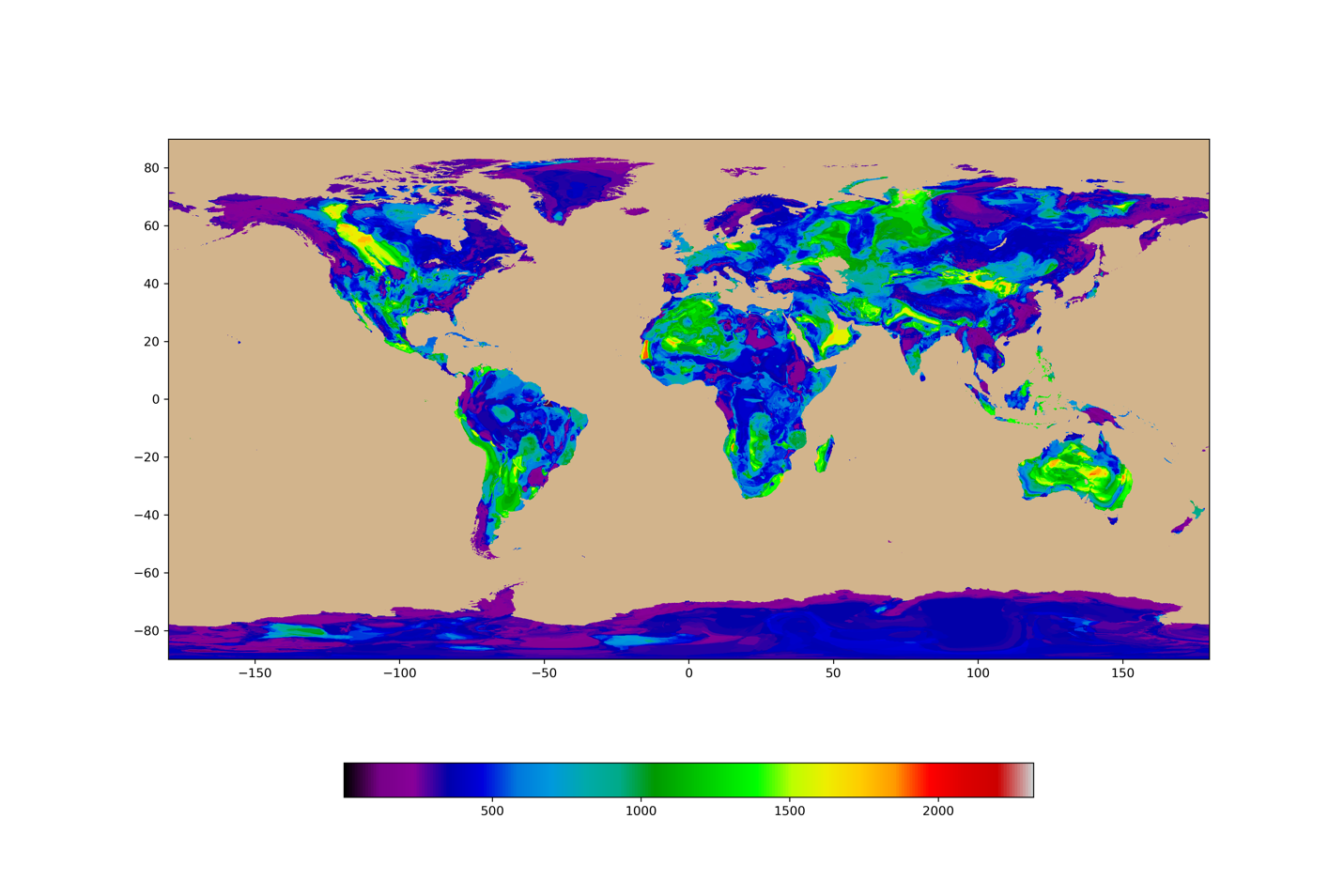


SI-2 Figure 12 -- GML map of groundwater specific conductance, in uS/cm


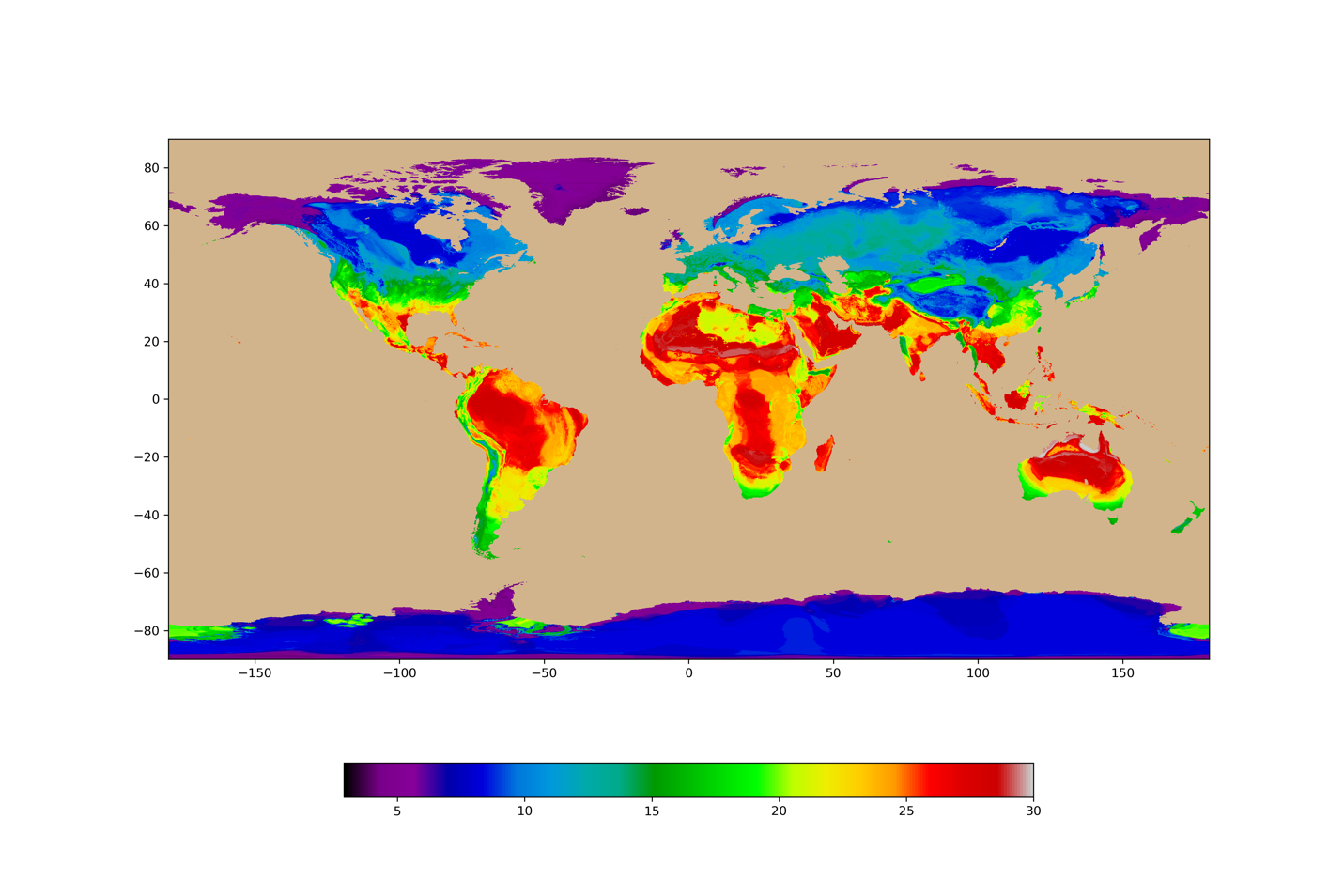


SI-2 Figure 13 -- GML map of groundwater temperature, in °C.


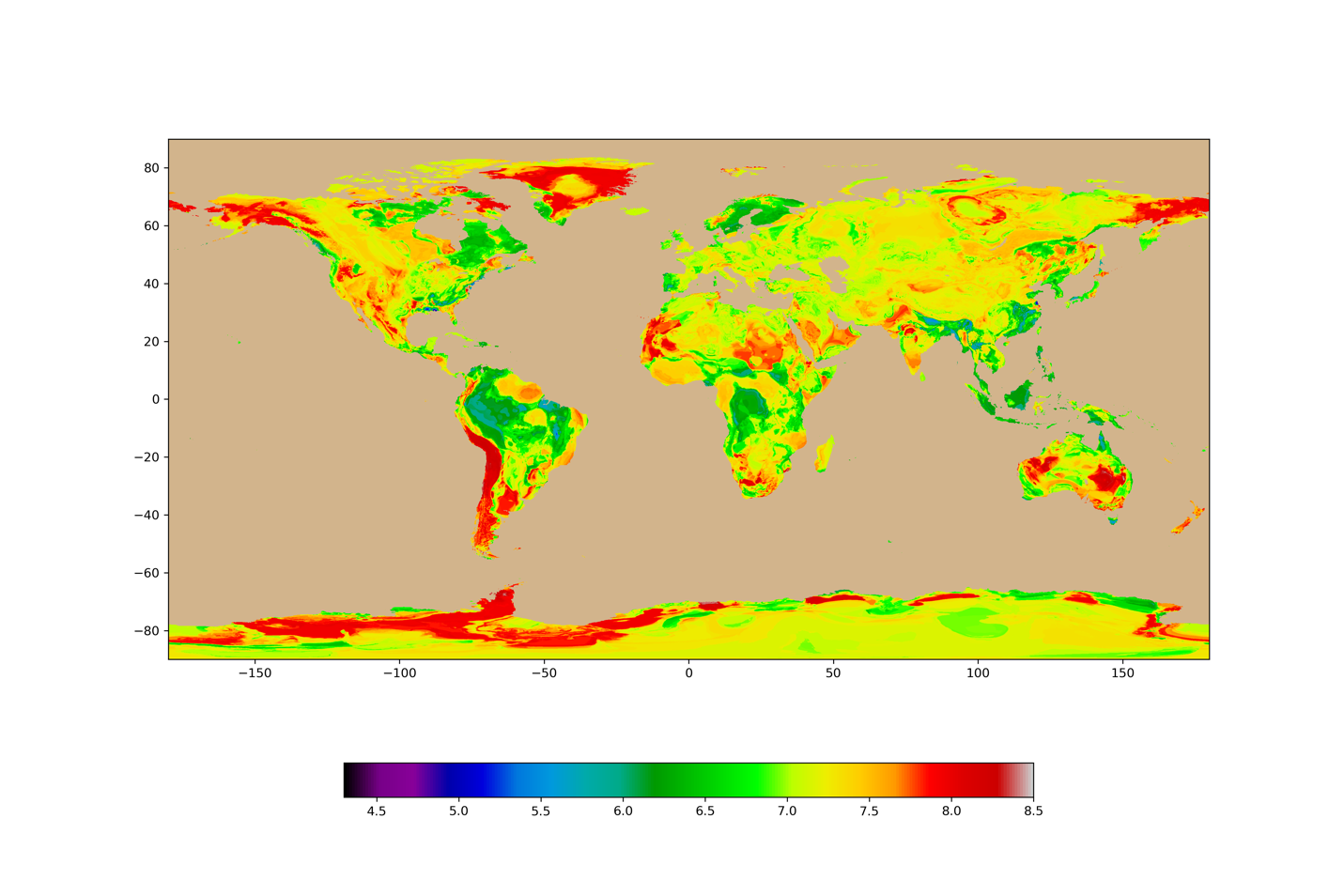


SI-2 Figure 14 -- GML map of groundwater pH, in pH units


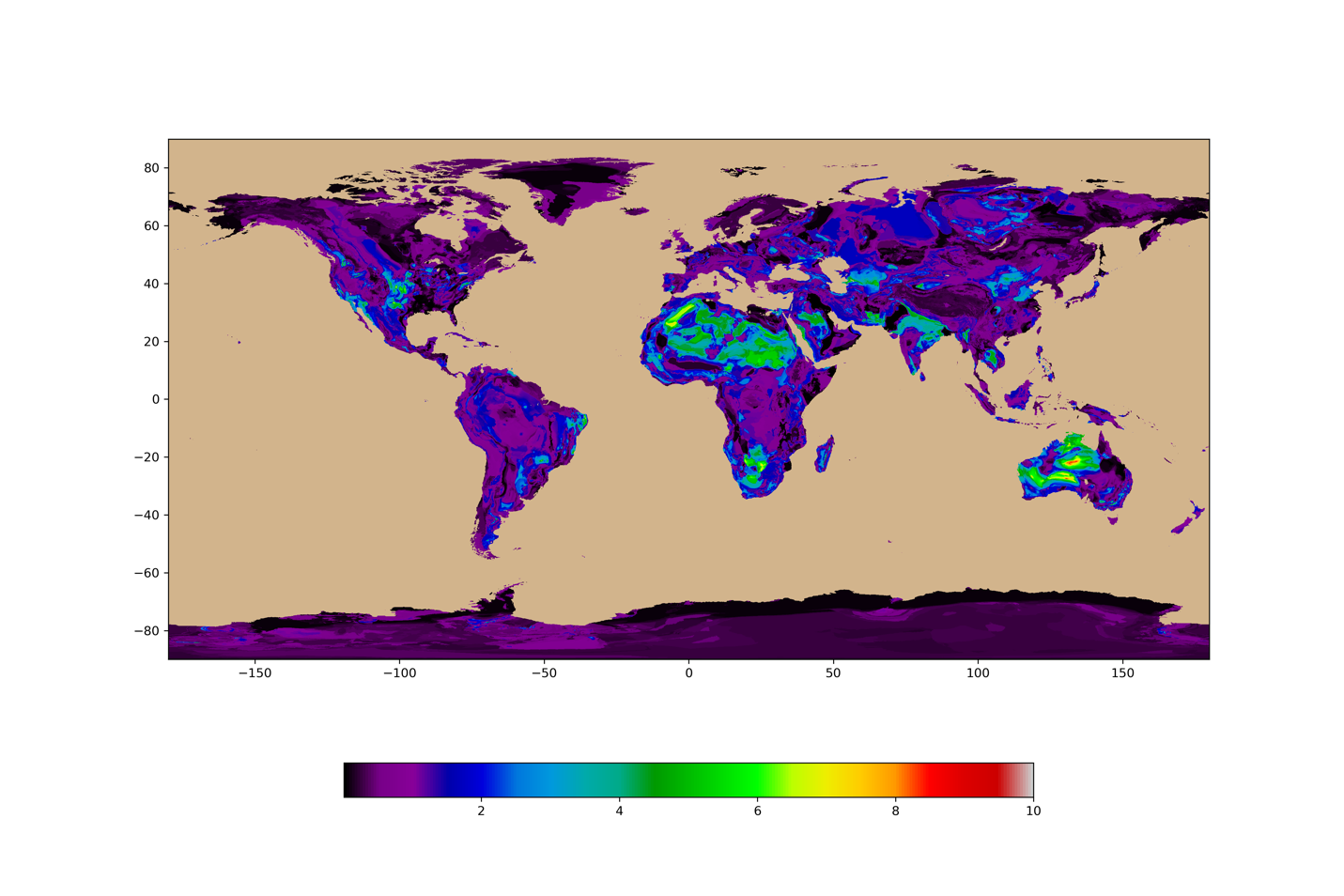


SI-2 Figure 15 -- GML map of groundwater NO_3_^-1^ as N, in mg/L

**Author address and contact information**

^1^Warren W. Wood,

Visiting Professor, Department of Earth and Environmental Sciences,

206 Natural Sciences Building,

288 Farm Lane,

Michigan State University,

East Lansing, Michigan 48824

USA

<[wwwood@msu.edu](mailto:wwwood@msu.edu)> corresponding author

^2^Pauline L. Smedley,

Hydrogeochemist,

British Geological Survey.

Nicker Hill,

Keyworth.

Nottingham, NG12 5GG,

UNITED KINGDOM

^3^Bruce D. Lindsey,

Groundwater Status and Trends Coordinator

U.S. Geological Survey

215 Limekiln Road

New Cumberland, PA 17070

USA

^4^Warren T. Wood

Geophysicist. Head, Geology and Geophysics Section

Naval Research Laboratory,

NRL Code 7432

John C. Stennis Space Center, MS 39529

USA

^5^Roberto E. Kirchheim –

National Coordinator of the Program for Isotope and Hydrochemistry

Geological Survey of Brazil (CPRM‐SGB)

Rua Costa, 55 - Cerqueira César
São Paulo - SP - Brazil
CEP: 01304-010

BRAZIL

^6^John A. Cherry,

Professor and Associate Director of Morwick G360 Institute for Groundwater Research,

University of Guelph,

50 Stone Road East, Thornbrough Building,

Guelph, Ontario, N1G 2W1

CANADA
